# Characterization of two novel proteins involved in mitochondrial DNA anchoring in *Trypanosoma brucei*

**DOI:** 10.1371/journal.ppat.1011486

**Published:** 2023-07-17

**Authors:** Simona Amodeo, Irina Bregy, Anneliese Hoffmann, Albert Fradera-Sola, Mara Kern, Hélène Baudouin, Benoît Zuber, Falk Butter, Torsten Ochsenreiter

**Affiliations:** 1 Institute of Cell Biology, University of Bern, Bern, Switzerland; 2 Graduate School for Cellular and Biomedical Sciences, Bern, Switzerland; 3 Institute for Anatomy, University of Bern, Bern, Switzerland; 4 Quantitative Proteomics, Institute of Molecular Biology GmbH, Mainz, Germany; Heidelberg University, GERMANY

## Abstract

*Trypanosoma brucei* is a single celled eukaryotic parasite in the group of the Kinetoplastea. The parasite harbors a single mitochondrion with a singular mitochondrial genome that is known as the kinetoplast DNA (kDNA). The kDNA consists of a unique network of thousands of interlocked circular DNA molecules. To ensure proper inheritance of the kDNA to the daughter cells, the genome is physically linked to the basal body, the master organizer of the cell cycle in trypanosomes. The connection that spans, cytoplasm, mitochondrial membranes and the mitochondrial matrix is mediated by the Tripartite Attachment Complex (TAC). Using a combination of proteomics and RNAi we test the current model of hierarchical TAC assembly and identify TbmtHMG44 and TbKAP68 as novel candidates of a complex that connects the TAC to the kDNA. Depletion of TbmtHMG44 or TbKAP68 each leads to a strong kDNA loss but not missegregation phenotype as previously defined for TAC components. We demonstrate that the proteins rely on both the TAC and the kDNA for stable localization to the interface between these two structures. *In vitro* experiments suggest a direct interaction between TbmtHMG44 and TbKAP68 and that recombinant TbKAP68 is a DNA binding protein. We thus propose that TbmtHMG44 and TbKAP68 are part of a distinct complex connecting the kDNA to the TAC.

## Author summary

*Trypanosoma brucei* is a single celled eukaryote that is only distantly related to most other model organisms. It is important to study this group in order to understand which parts of their biology are conserved and which have diverged throughout evolution. *T*. *brucei* cells—like most eukaryotes—contain mitochondria. Trypanosomes however, only harbor one mitochondrion with a singular mitochondrial genome per cell. It has been shown that the mitochondrial genome in these organisms is physically linked to the base of the flagellum and that this linkage, which has been named Tripartite Attachment Complex (TAC), is required for proper distribution of the mitochondrial genome during cell division. In the current study we identify and characterize two novel components of a distinct complex involved in connecting the TAC to the mitochondrial DNA.

## Introduction

*Trypanosoma brucei* is a single celled parasite that belongs to the Kinetoplastea within the eukaryotic supergroup of the Excavates [1]. The distinguishing feature of this group is the organization of its single mitochondrial genome into a large structure called the kinetoplast [2,3]. In *T*. *brucei* the kinetoplast consists of 25 maxicircles that encode 18 protein genes and two ribosomal RNAs. Twelve of the 18 protein genes are cryptogenes that require post transcriptional editing involving a large enzymatic machinery (Editosome) and small trans-acting guide RNAs [4–8]. The guide RNAs are encoded on the 400 different minicircle species in the network that is coding for a total of 1300 gRNA genes [9]. Overall, there are 5000–10000 minicircles that are catenated with neighboring minicircles and the maxicircles to form the kinetoplast DNA (kDNA) [2,3]. Replication of the minicircles has been characterized in detail for *T*. *brucei* (for reviews see [10,11] and [12]). In brief, minicircles are released from the network and replicated via theta intermediates. After partial repair of the gaps, they are re-attached to the network where the remaining gaps are closed. Much less is known about the replication of the maxicircles but they likely remain attached to the network during this process. Currently, more than 30 proteins have been described to be involved in kDNA maintenance, including replication, while far fewer are required for this process in other eukaryotic systems [10–13]. After replication of the mini- and maxicircles is completed the kDNA network is segregated into the two daughter networks.

The kDNA anchoring and segregation machinery of *T*. *brucei*, named the Tripartite Attachment Complex (TAC), was discovered in 2003 and described by transmission electron microscopy [14]. The TAC consists of three regions spanning three compartments of the cell (i) the exclusion zone filaments (EZF) that range from the base of the flagellum to the outer mitochondrial membrane (ii) the differentiated mitochondrial outer and inner membranes (DM) and (iii) the unilateral filaments (ULF) that range from the inner mitochondrial membrane to the kDNA and can be further subdivided in a kDNA proximal, basic domain (pH) and an inner mitochondrial membrane proximal domain that is largely acidic in nature [14]. The assembly of the TAC occurs *de novo*, from the base of the flagellum towards the kDNA, in a hierarchical manner, such that kDNA proximal components depend on the proper assembly of the kDNA distal components [13,15]. The current model of the TAC includes 13 protein components [13]. Four of these are localized to the EZF (p197, BBA4, Mab22 and TAC65; [16–18]). The DM harbor four proteins of the TAC in the outer mitochondrial membrane (TAC60, TAC42, TAC40 and pATOM36, [18–20]) while one component (p166) is associated with the inner mitochondrial membrane [21,22]. p166 is a transmembrane protein in which the C-terminal region extends into the intermembrane space and connects to TAC60 of the OMM [23]. While the N-terminus of p166 is part of the ULF and connects to the N-terminus of TAC102 the kDNA most proximal candidate of the TAC [24]. The only other known protein component of the ULF is TAP110 [25]. While TAP110 is proximal to the kDNA when compared with TAC102, it does not seem to be essential for kDNA maintenance and thus might not be a core component of the TAC. There are also several additional components that—despite having multiple localizations—are likely part of the TAC. This includes for example the E2 subunit of the α-ketoglutarate dehydrogenase, the tubulin-binding cofactor C protein and AEP1 in the inner mitochondrial membrane [21,22,26,27]. Aside from the proteins involved in the TAC there are also several proteins that likely play a role in organization of the kDNA network and its anchoring to the TAC. These include the kDNA associated proteins KAP3, KAP4 and KAP6. KAP3 was described as a histone H1 like protein in *Crithidia fasciculata*, while KAP6 is an HMG-box containing protein described in *T*. *brucei* [28,29].

In this study we identify two novel proteins responsible for linking the kDNA to the TAC and characterize the organization of this region which has previously been shown to primarily consist of basic proteins [30]. We develop a new approach combining flagellar extraction with RNAi and quantitative proteomics to analyse the dependencies in this otherwise mainly insoluble structure. Based on the localization, biochemical properties and their requirement for DNA to be present for their assembly, we propose that the two proteins are part of a distinct complex that connects the TAC and the kDNA.

## Results

### TbmtHMG44 and TbKAP68 are interaction partners of TAC102

In an attempt to identify novel interaction partners of TAC102 that could provide a connection to the kDNA we expressed a YFP tagged version of TAC102 in PCF cells (S1 Fig), isolated cytoskeletons, sonicated the sample and immunoprecipitated TAC102 using a GFP specific antibody. We identified 1523 proteins and among the top 50 enriched proteins, 21 were predicted/annotated to be mitochondrion or axoneme associated (S1 Table). This included six already characterized TAC components: TAC102, TAP110, p166, TAC60, TAC65, and TAC40 [18–20,22,24,25]. Also, among the top 50 enriched proteins we detected the two very basic, potentially essential proteins, Tb927.9.5020 and Tb927.11.16120 that were predicted to be kinetoplast and mitochondrially localized, respectively (S1 Table) [31]. Since the inner unilateral filaments between TAC102 and the kDNA have been proposed to be largely basic in nature (see introduction) we decided to characterize Tb927.9.5020 and Tb927.11.16120 in more detail.

Tb927.11.16120 is a basic (pI = 10.0), 68 kDa protein with a predicted mitochondrial targeting sequence at the N-terminus [32]. The protein was previously identified as component of the mitochondrial importome [33]. In response to the findings presented in this study, we chose to refer to Tb927.11.16120 as TbKAP68 (kDNA associated protein of a size of 68kDa).

Tb927.9.5020 is a basic (pI 10.2), 44 kDa protein. It has a putative HMG-box domain at the N-terminus and thus we will refer to Tb927.9.5020 as TbmtHMG44.

Both proteins are present in the majority of the currently sequenced Kinetoplastea genomes. Neither of the candidates is represented in the genome of *Perkinsela species*. Perkinsela is an endosymbiotic kinetoplastid that lacks basal body and flagellum [34]. Interestingly, we can readily identify a homologue of TbKAP68 in the genome of *Bodo saltans* (CU90103.1), while no homologue of TbmtHMG44 was found in the free-living kinetoplastid [35].

### TbKAP68 and TbmtHMG44 localize in the unilateral filament region of the TAC and are closer to the kDNA than TAC102

**Fig 1:**
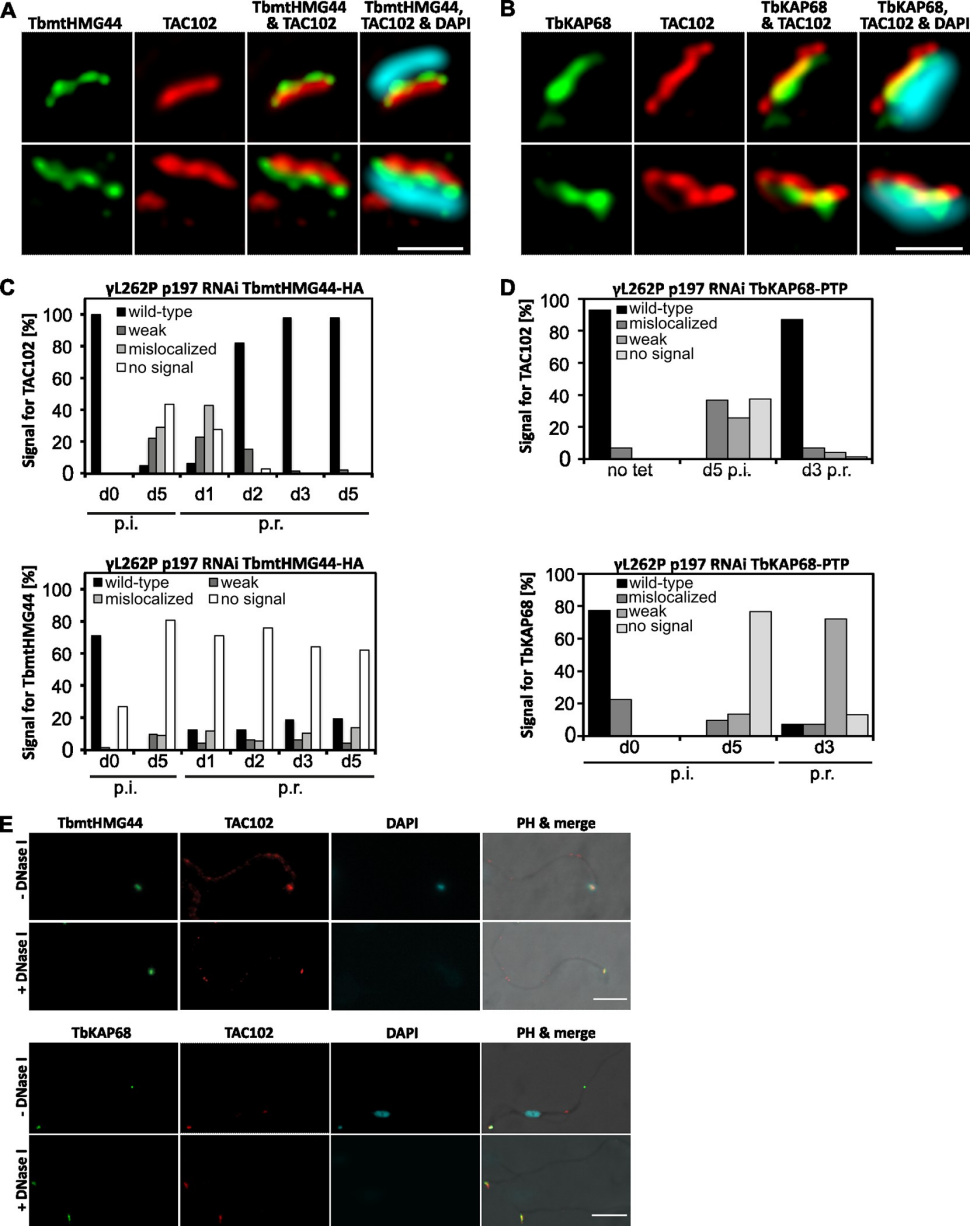
TAC and kDNA are required for localization of TbmtHMG44 and TbKAP68 to the interface between TAC102 and the kDNA. A) 2D-STED immunofluorescence microscopy of TbmtHMG44-HA relative to TAC102 (monoclonal anti-TAC102 antibody) and the kDNA (DAPI, acquired using confocal microscopy). Scale bar 500 nm. B) 2D-STED immunofluorescence microscopy of TbKAP68-PTP relative to TAC102 and the kDNA. Localization dynamics of TbKAP68 across the cell cycle are depicted in S2 Fig. Scale bar 500 nm. C) Quantitative analysis of the fluorescent signals for TAC102 (top) and TbmtHMG44-HA (bottom) relative to the basal body marker YL1/2, over the course of depletion and reassembly of the TAC. (exemplary images from widefield fluorescence microscopy in S3A Fig; quantification of kDNA loss in S3C Fig) n ≥ 100 cells D) Quantitative analysis of the fluorescent signals for TAC102 (top) and TbKAP68-PTP (bottom) relative to the basal body marker YL1/2, over the course of depletion and reassembly of the TAC (exemplary images from widefield fluorescence microscopy in S3B Fig; quantification of kDNA loss in S3D Fig) n ≥ 100 cells. p.i., post induction; p.r.; post recovery. E) Microscopic analysis of TbmtHMG44 (top) or TbKAP68 (bottom) on isolated flagella (widefield fluorescence microscopy; immunodetection of HA or PTP epitope tags and TAC102, detection of DNA with DAPI, visualization of flagella with phase contrast (PH)). Flagella are either DNase I treated or untreated Scale bars 5 μm.

To determine the subcellular localization of TbKAP68 and TbmtHMG44, we generated *in situ* tagged cell lines in BSF cells (TbKAP68-PTP and TbmtHMG44-HA). The respective protein tags and the known TAC component TAC102 were visualized using immunofluorescence analysis (IFA) and stimulated emission depletion (STED) microscopy (Fig 1). We observed both proteins to localize in close proximity to the kDNA (Fig 1A and 1B). While the signal for TbmtHMG44-HA localized in between TAC102 and the kDNA, without overlapping with either of them, the signal for TbKAP68 often overlapped with the TAC102 signal. Despite the signal overlap, TbKAP68 was localizing closer to the kDNA than TAC102 overall. When tracking the signal for TbKAP68-PTP across cells of different cell cycle stages, we observed that the signals separate later in cell cycle than the TAC102 signal does (S2 Fig, arrowheads). All together, these observations suggest that both TbmtHMG44 and TbKAP68 localize closer to the kDNA than TAC102 does.

### TbmtHMG44 and TbKAP68 remain stably associated with the TAC in DNase I treated flagella but not in dyskinetoplastic cells

To assess the interactions of TbKAP68 and TbmtHMG44 with the surrounding structures (the TAC and the kDNA), we created cell lines in which TAC assembly is reversibly inhibited. As shown before by others and us, the depletion of p197 by RNAi leads to disruption of the TAC, ultimately resulting in kDNA loss and cell death [15]. However, in the previously described mutant BSF cell line (γL262P), the depletion of p197 leads to disruption of the TAC without significant impact on fitness of the cells, as they are able to proliferate without the kDNA [36]. This allows us to reversibly disrupt TAC biogenesis and create dyskinetoplastic cells. For our study we created BSF p197 RNAi TbmtHMG44-HA and p197 RNAi TbKAP68-PTP cell lines in the γL262P background. In IFA widefield microscopy we observed that upon p197 RNAi induction, the signals for TbmtHMG44 and TbKAP68 disappear (Fig 1C and 1D, bottom; representative images in S3A and S3B Fig). At day five of p197 RNAi we verified that all cells had lost their kDNA (S3C and S3D Fig). We then allowed re-expression of p197, and observed that, while TAC102 restores to wild type localization within three days (Fig 1C and 1D, top), TbmtHMG44 and TbKAP68 do not recover wild type localizations in dyskinetoplastic cells, even after five days after release from p197 RNAi (Fig 1C and 1D bottom; representative images in S3A and S3B Fig). This suggests that TbmtHMG44 and TbKAP68 rely on the kDNA for proper assembly to their wild type localization, while known TAC components like TAC102 re-assemble into the TAC without the mitochondrial genome *in vivo*.

In a next step we analyzed if TbKAP68 and TbmtHMG44 require DNA to remain associated with the TAC (Fig 1E). For this we isolated flagella from TbKAP68-PTP and TbmtHMG44-HA cells. We then either treated the isolated flagella with DNase I, or fixed them without further treatment. From both sample types we performed IFA microscopy. Interestingly, the experiments show that both proteins associate with isolated flagella, even after DNase I treatment (Fig 1E). This suggests that, once assembled, the two proteins do not require the kDNA for proper localization anymore. In combination with the TAC depletion/recovery experiment, these results suggest a kDNA dependent assembly of these proteins, followed by their anchoring onto a structure other than the DNA itself.

### Depletion of TbKAP68 or TbmtHMG44 leads to kDNA loss

**Fig 2:**
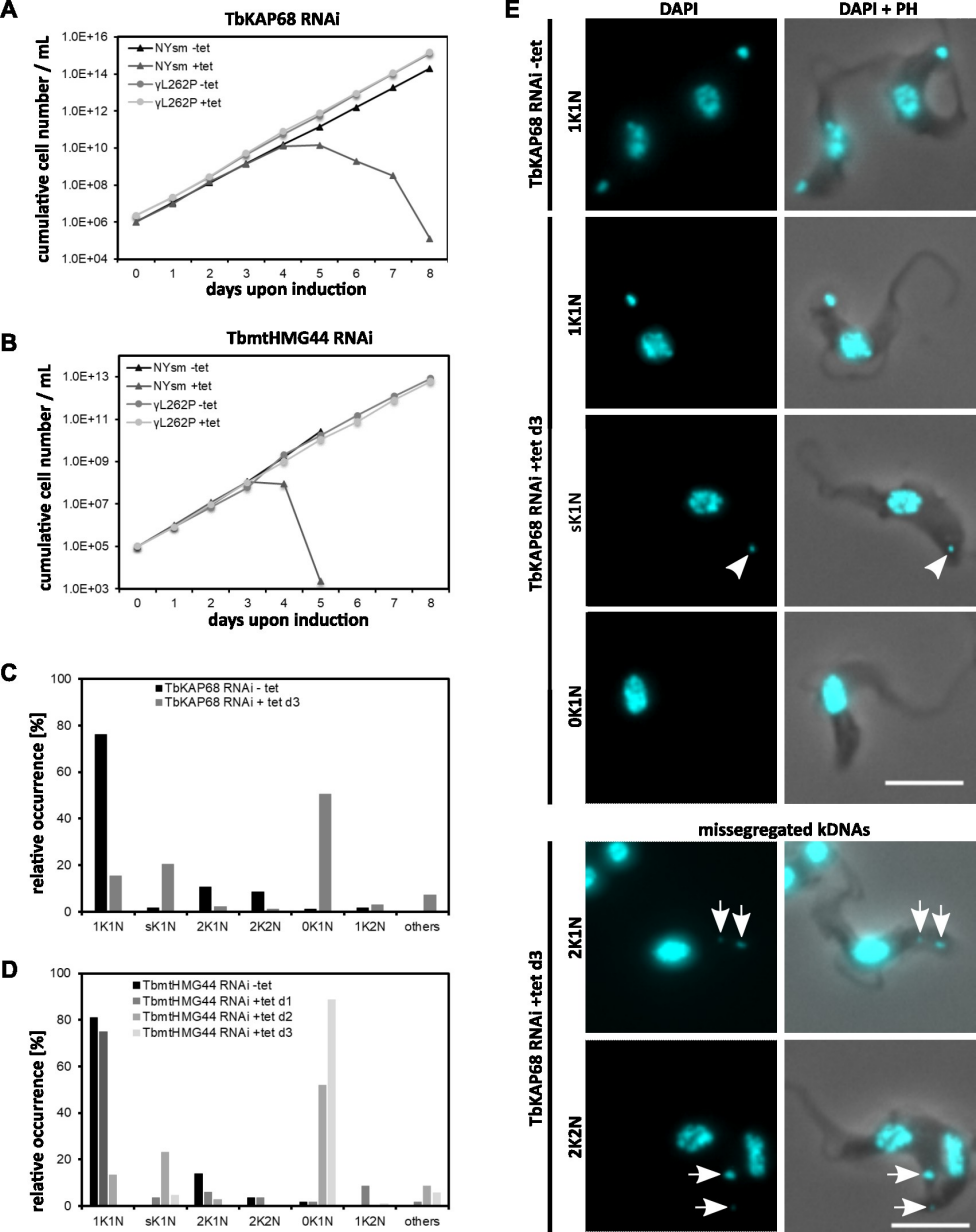
TbKAP68 and TbmtHMG44 are essential for survival and kDNA maintenance. A) Growth curves of NYsm or γL262P BSF *T*. *brucei* cells expressing TbKAP68 RNAi. Knockdown efficiency in NYsm was quantified by northern blot (63% signal loss on day 3, S4C Fig). B) Growth curves of NYsm or γL262P BSF *T*. *brucei* cells expressing TbmtHMG44 RNAi. Knockdown efficiency in NYsm was evaluated by quantitative reverse transcription PCR (73% depletion on day 3, S4B Fig). C) Quantification of kDNA shrinkage and loss after three days TbKAP68 RNAi induction in NYsm (N ≥ 150). Numbers of kDNAs (K) and nuclei (N) per cell were counted based on the DAPI signal (DNA) and phase contrast (cell body) and listed accordingly. sK1N cells contain a single kDNA that is clearly smaller than the wild type kDNA network. (corresponding graph for γL262P TbKAP68 RNAi cells shown in S4D Fig) D) Quantification of kDNA shrinkage and loss after one, two and three days of TbmtHMG44 RNAi in NYsm(N ≥ 100). (corresponding graph for γL262P TbmtHMG44 RNAi cells shown in S4E Fig) E) Representative images of phenotypic appearance of NYsm TbKAP68 RNAi cells at day zero and day three post RNAi induction. Arrowheads point at the small kDNA of an sk1N cell. Arrows point at the two kDNAs of cells with missegregated kDNA networks. Scale bar 5 μm. (corresponding imagery showing the phenotype of NYsm TbmtHMG44 RNAi shown in S4A Fig).

To study the function of TbKAP68 and TbmtHMG44, we performed RNAi targeting the open reading frames of the mRNAs in BSF cells (cell lines TbKAP68 RNAi and TbmtHMG44 RNAi, knockdown efficiency demonstrated in S4B and S4C Fig, mRNAs were decreased by 70% in both cases). Depletion of either TbKAP68 or TbmtHMG44 resulted in a growth defect five and four days post induction, respectively (Fig 2A and 2B). In order to test if the growth defect is due to a function in kDNA maintenance or mitochondrial gene expression, we made use of the γL262P cell line once again. Using γL262P TbKAP68 RNAi and γL262P TbmtHMG44 RNAi, we demonstrate that neither of these cell lines shows a growth defect even after 8 days of RNAi induction (Fig 2A and 2B). We thus conclude that both, TbKAP68 and TbmtHMG44 are involved in kDNA related processes only.

We also characterized the effect of TbKAP68 and TbmtHMG44 depletion on the kDNA. For this we applied DAPI staining and widefield fluorescence microscopy on the NYsm RNAi cell lines of both candidates in uninduced state, as well as three days post RNAi induction (Figs 2C–2E, S4A, S4D and S4E). In TbKAP68 RNAi as well as TbmtHMG44 RNAi, we observed a strong accumulation of cells without kDNA. At day three post RNAi induction the number of cells without kDNA reached 50% and 90% in the TbKAP68 and TbmtHMG44 RNAi cells, respectively. Furthermore, we observed an increasing number of cells with smaller kDNAs in both RNAi cell lines (Fig 2C–2E sk1N cells). In case of TbKAP68 depletion we detected a small number of cells with missegregated kDNAs (Fig 2E bottom panel). Altogether, the results suggest both proteins are involved in kDNA maintenance. The observation of missegregated kDNAs for TbKAP68 RNAi, could suggest that the protein is also involved in kDNA segregation.

### Effect of TbmtHMG44 and TbKAP68 depletion on kDNA network composition

**Fig 3:**
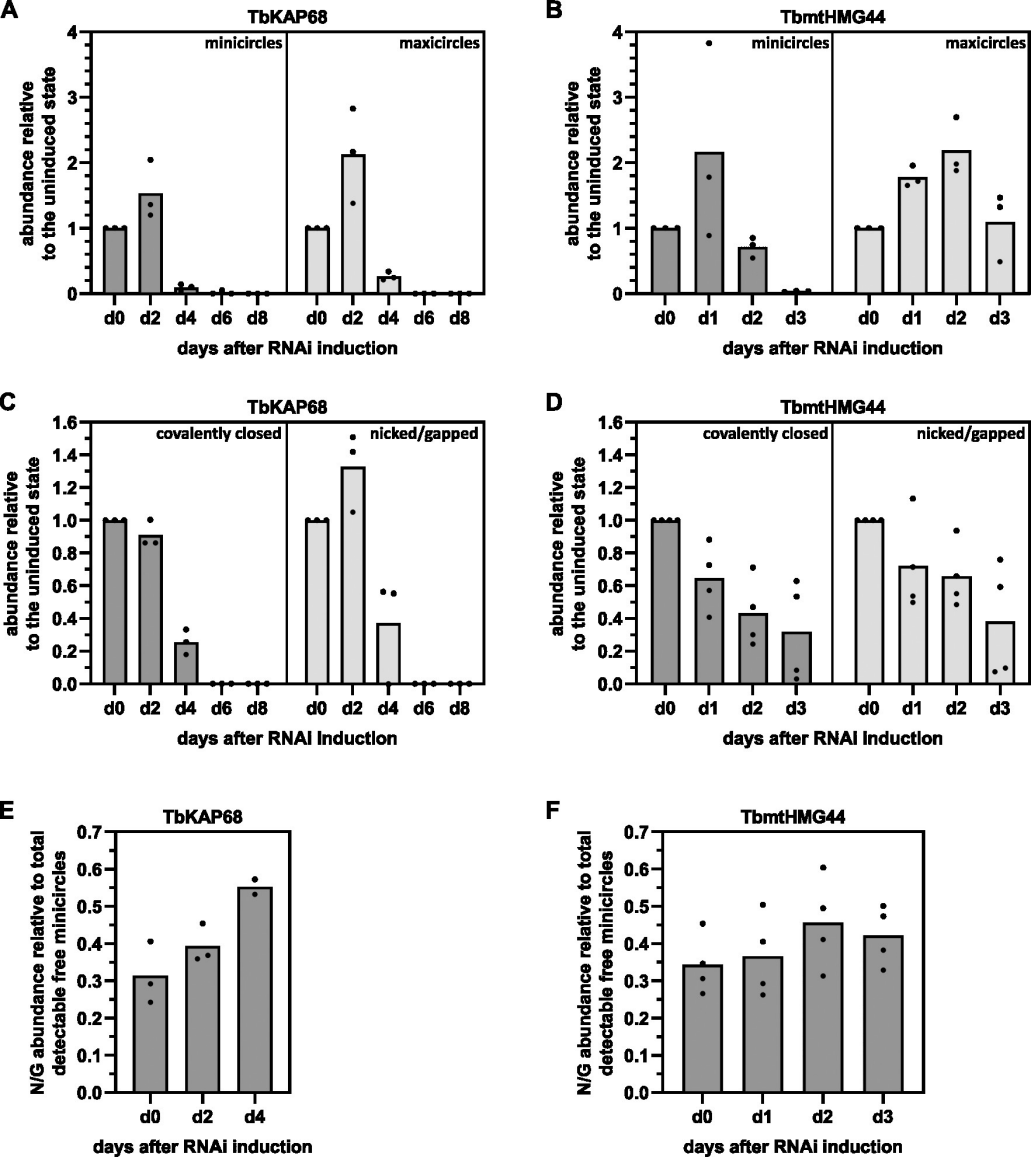
TbKAP68 and TbmtHMG44 depletion has different effects on the kDNA. A) Dynamics of the relative abundance of total minicircle and total maxicircle content upon depletion of TbKAP68. Mini- and maxicircle abundance were measured using Southern blot analysis of total DNA from TbKAP68 RNAi cells. The DNA was digested with HindIII and XbaI to linearize kDNA molecules prior to gel electrophoresis. The signals on the Southern blot were normalized by probing for α-tubulin on the same blot. The experiment was performed in triplicates (individual values shown). The mean of the three data points for each time point is represented by the height of the grey bar in the respective column. B) Dynamics of the relative abundance of total minicircle and total maxicircle content upon depletion of TbmtHMG44. The experimental procedure was identical to A. The increase of the maxicircle signal from day 0 to day 1 or day 2, respectively was rated as significant based on a two-sided, paired t-test (p-values of 0.014 or 0.044, respectively). C) Dynamics of the relative abundance of minicircle replication intermediates upon depletion of TbKAP68. Southern blot analysis was performed using undigested total DNA of TbKAP68 RNAi cells. Signals of free minicircle replication intermediates were normalized to α-tubulin. The graph depicts covalently closed, unreplicated free minicircles, as well as nicked/gapped freshly replicated free minicircles. The experiment was performed in triplicates (individual values shown). The mean of the three data points for each time point is represented by the height of the grey bar in the respective column. D) Dynamics of the relative abundance of minicircle replication intermediates upon depletion of TbmtHMG44. The experimental procedure was identical to C. E) Quantification of the relative proportions of nicked/gapped minicircles in respect to the total of free minicircles (consisting of the total of nicked/gapped and covalently closed minicircles) detected at the same time point of TbKAP68 RNAi. The mean of the individual data points for each time point are represented by the height of the grey bar in the respective column. F) Quantification of the relative proportions of nicked/gapped minicircles in respect to the total of free minicircles (consisting of the total of nicked/gapped and covalently closed minicircles) detected at the same time point of TbmtHMG44 RNAi. Examples of each type of Southern blot performed for this figure are depicted in S5 Fig.

Since the depletion of TbmtHMG44 and TbKAP68 led to kDNA loss, we wanted to further characterize the effect on network structure, the different DNA types (mini- and maxicircles) and minicircle replication intermediates (covalently closed (CC) circles prior to replication vs nicked and gapped (N/G) circles just after replication). To assess the effect on mini- and maxicircles, we used Southern blot analysis. We extracted total DNA from the γL262P TbmtHMG44 RNAi and the γL262P TbKAP68 RNAi cell lines and treated it with the restriction enzymes HindIII and XbaI. We then resolved the digested DNA on an agarose gel, transferred it to a membrane and probed for mini-and maxicircles (Southern blots in S5A and S5B Fig). The amount of minicircles increased to about 150% at day two of TbKAP68 depletion and the maxicircle content even increased to around 210% (Fig 3A). Only after this initial increase, the levels of both, mini- and maxicircles dropped drastically and were undetectable by day six of RNAi induction. Depletion of TbmtHMG44 let to an increase of minicircle content at day one only, while at day two, minicircle content decreased to around 70% of the uninduced level (Fig 3B). For the maxicircles a similar increase to around 220% was observed at day two post RNAi induction. At day three of TbmtHMG44 RNAi we observed a strong decrease of minicircles, while the maxicircle content remained elevated (around 180%).

Free minicircle replication intermediates released for replication (CC and N/G) can be analyzed by Southern blot when using total, non-digested DNA from the RNAi cell lines (Southern blots in S5C and S5D Fig). At day two of TbKAP68 RNAi, the CC, not yet replicated, minicircles were detected with almost the same abundance as in non-induced cells, whereas at day four of the RNAi the CC level had dropped to about 20% (Fig 3C). The N/G (replicated but not reattached) minicircles in contrast, increased to around 130% at day two of TbKAP68 depletion, before the abundance rapidly decreased to around 40% at day four of the RNAi. The amount of N/G minicircles relative to the total free minicircle content (N/G + CC), gradually increased over the course of TbKAP68 RNAi induction (Fig 3E). Knockdown of TbmtHMG44 led to a constant loss of CC and N/G minicircles (around 40% of CC and N/G were left at day three post induction) (Fig 3D). The ratio between CC and N/G minicircles upon TbmtHMG44 knockdown did not show a trend towards either replication intermediate (Fig 3F). In conclusion we can state that depletion of TbKAP68 leads to concomitant loss of mini and maxi circles, while depletion of TbmtHMG44 only seems to affect the minicircle abundance. The minicircle replication intermediates largely follow this trend, however it seems that the relative number of replicated molecules slightly increases or remains unchanged suggesting that replication itself is still at least partially functional.

### Direct interactions between TbKAP68 and TbmtHMG44 *in vitro*

**Fig 4:**
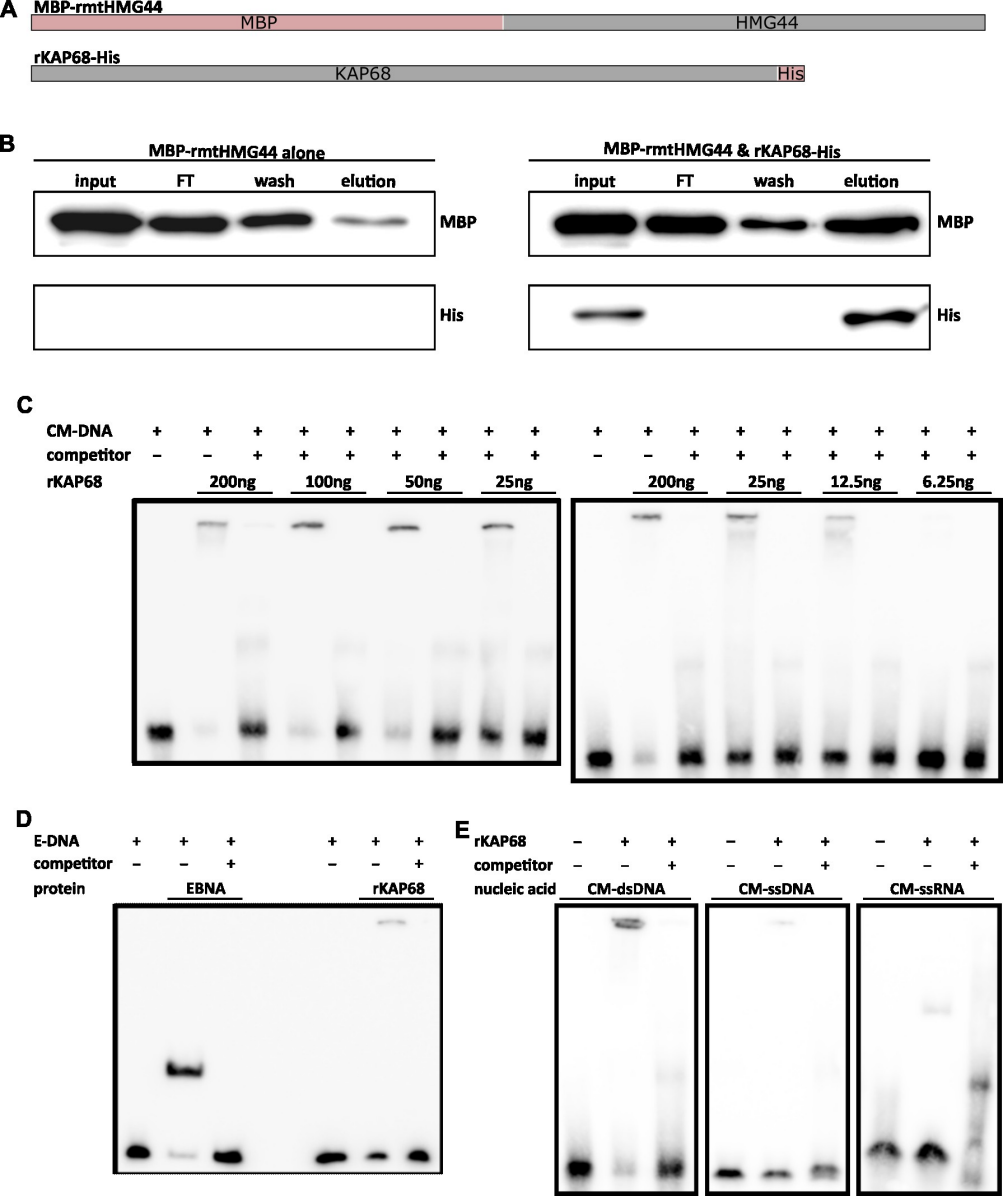
**In vitro binding assays show interactions of TbKAP68 with TbmtHMG44 and pure DNA probes.** A) Schematic overview of the constructs used for the experiments in B-F. MBP-rmtHMG44 consists of the native TbmtHMG44 sequence obtained by PCR of 427 strain *T*. *brucei* DNA isolate, and an N-terminal MBP tag. (S6A and S6B Fig show samples of the purified recombinant proteins on SDS-PAGE) B) Co-affinity precipitation experiment with MBP-rmtHMG44 and rKAP68-His. In the negative control experiment (left), MBP-rmtHMG44 was was incubated on nickel resin without the addition of rKAP68-His. The blot on the right was generated from a sample containing MBP-rmtHMG44 and rKAP68-His. Both recombinant proteins were mixed and incubated on Ni-NTA resin. In both experiments, the resin were washed 5 times, and bound proteins were eluted from the resin. The His tag of rKAP68-His has nickel binding affinity and thus directly interacts with the resin. MBP-rmtHMG44 does not bind the resin (experiment on the left). MBP-rmtHMG44 was monitored on SDS-PAGE western blot probed with anti-MBP antibody (top row). rKAP68-His was tracked on the same blot, using an anti-His antibody (bottom row). The experiment was performed in quatruplicates (additional replicates in S6C Fig; similar experiment using rKAP68-ΔN-His shown in S6D Fig). C) Titration of purified recombinant KAP68 protein expressed with a 6x Histidine tag (rKAP68-His) to determine minimum amount of protein needed to shift DNA. We used a biotinylated, conserved minicircle DNA (CM-DNA) as a bait. 200-fold excess of the same but non-biotinylated DNA were used as competitor (C). D) Left side: EMSA using biotinylated EBNA DNA (E-DNA and an EBNA nuclear protein extract. 200-fold excess of the same DNA, but non-biotinylated, was used as competitor. Right side: EMSA using the same EBNA DNA but 200ng purified rKAP68-His protein. E) EMSAs using either single-stranded DNA or RNA as a bait. 200ng of rKAP68-His were used.

Based on the information that TbKAP68 and TbmtHMG44 localize very close to each other (Fig 1) and that the two proteins behave similar in many of the experiments described above, we suspected the two proteins to be direct interactors. In order to test this hypothesis, we purified recombinant versions of both proteins (TbmtHMG44 with an N-terminal MBP tag (MBP-rmtHMG44) and TbKAP68 with a C-terminal 6xHis tag (rKAP68-His)) from *Escherichia coli* (Figs 4A, S6A and S6C). The purified proteins were mixed in a 1:1 molar ratio, incubated and then loaded on Ni-NTA resin (MBP-rmtHMG44 alone was loaded in the control). We followed MBP-rmtHMG44 by SDS-PAGE and western blot analysis using an anti-MBP antibody and could show the interaction of the two proteins *in vitro* (Figs 4B and S6C). To localize the TbmtHMG44 binding domain of TbKAP68, we performed the same experiment with the C-terminal half of TbKAP68 (S6D Fig; rKAP68-ΔN-His construct in S6A Fig). We mixed MBP-rmtHMG44 with rKAP68-ΔN-His in a 1:1 molecular ratio, and performed the experiment as described previously. Our results demonstrate that MBP-rmtHMG44 binds to rKAP68-ΔN-His (S6D Fig, top), while not binding to the resin directly (control panel S6D Fig, bottom). We thus conclude that for *in vitro* interactions with TbmtHMG44, the TbKAP68 C-terminal half is sufficient.

### Recombinant TbKAP68 binds without sequence specificity to DNA *in vitro*

Due to their vicinity to the kDNA, we wanted to test whether TbKAP68 and/or TbmtHMG44 bind to DNA. For this, we performed electrophoretic mobility shift assays (EMSAs). We used a 73bp stretch of the conserved minicircle region as a bait and purified recombinant MBP-rmtHMG44 or rKAP68-His protein to analyze their respective nucleic acid binding abilities (purifications shown in S6A and S6B Fig; constructs shown in Fig 4A). We detected a shift of the DNA to the top of the gel when 200 ng of rKAP68-His was added to a reaction containing 20 fmol of DNA bait (Fig 4C). By adding 200-fold excess of the same non-biotinylated DNA we were able to compete for binding to the biotinylated probe. To determine the minimal amount of rKAP68-His required to obtain a shift of the DNA, we performed a titration with 200ng, 100ng, 50ng, 25ng and 6.25ng. With 200ng and 100ng of rKAP68-His almost all DNA was shifted, while the shifted amounts of DNA dropped gradually when using amounts as small as 50ng, 25ng and 6.25ng. To test whether TbKAP68 binds to DNA in a sequence specific manner, we also performed an EMSA with rKAP68-His using the Epstein-Barr Nuclear Antigen (EBNA) control DNA as a bait (Fig 4D). The control reaction with EBNA DNA in combination with EBNA extract showed that the conditions applied in our experiments enable efficient binding of EBNA DNA by a suitable binding partner (EBNA extract). When using rKAP68-His to shift EBNA DNA we also detected a shift. Just like in the previous setting, rKAP68-His shifted the bait to the top of the gel. We conclude that, under the conditions tested, rKAP68-His binds to double stranded DNA without any obvious sequence specificity. When offering single stranded DNA or RNA as a bait instead, only a small portion of the bait was shifted (Fig 4E). In case of an RNA bait, the migration behavior changed so that small amounts of bait shifted to create a band that was running lower in the gel than was observed for DNA baits. From these results we conclude that recombinant TbKAP68 primarily binds double stranded DNA in the *in vitro* setting applied here. We did not observe any DNA binding activity for TbmtHMG44 under the conditions tested (S6E and S6F Fig).

### The HMG-box domain of TbmtHMG44 is important for correct localization of the protein

**Fig 5:**
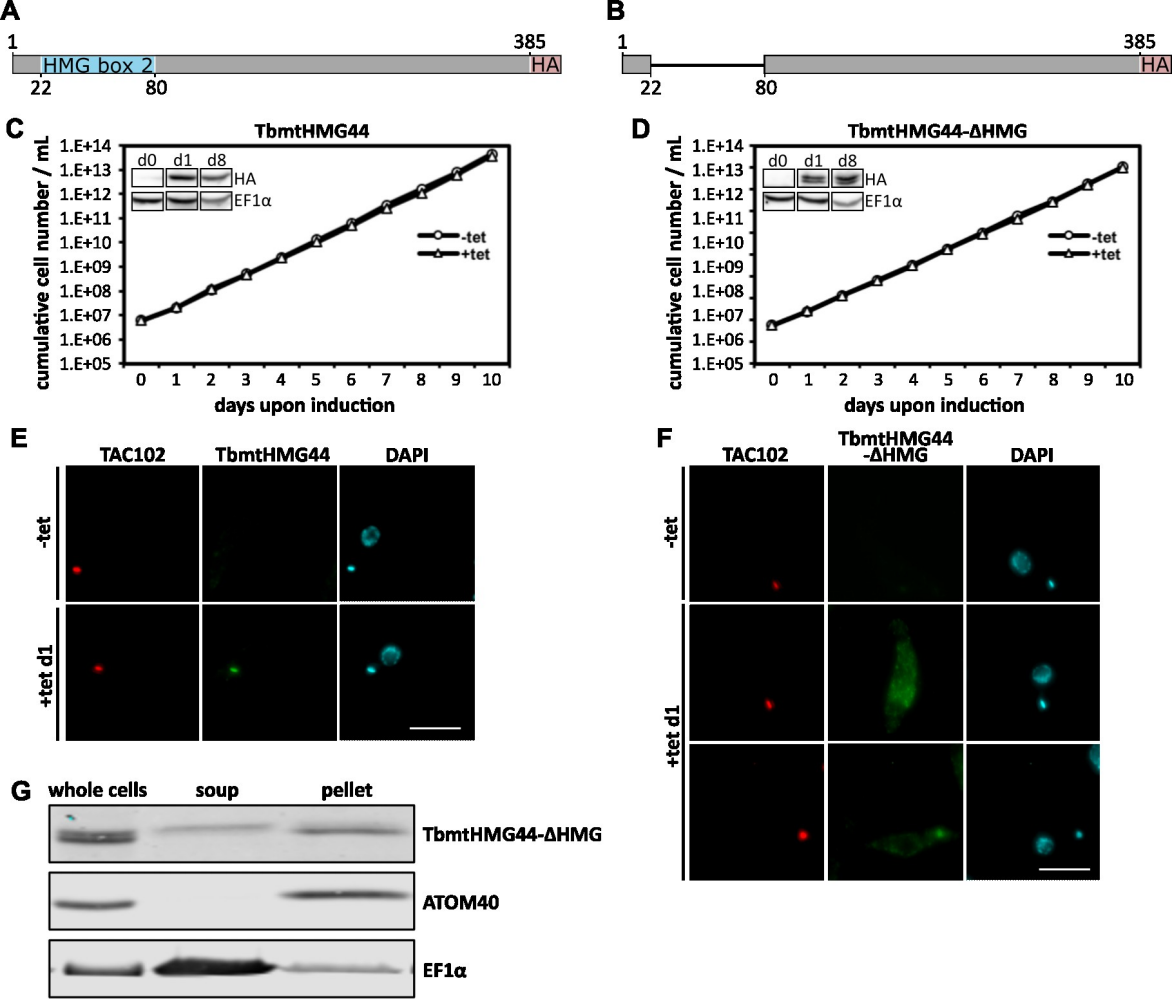
Expression of TbmtHMG44-HA and TbmtHMG44-ΔHMG-HA in PCF cells. A) Schematic overview of the TbmtHMG44-HA protein sequence with the HMG-box highlighted in blue and the triple HA-tag highlighted in red. B) Depiction of the TbmtHMG44-ΔHMG-HA protein sequence with the triple HA-tag highlighted in red. C) Growth curve of cells induced for TbmtHMG44-HA overexpression. The Inset shows a western blot of the TbmtHMG44-HA overexpression cell line at days zero, one and eight of construct expression. EF1α serves as loading control. D) Growth curve of TbmtHMG44-ΔHMG-HA overexpression. The Inset shows a western blot of the TbmtHMG44-ΔHMG-HA overexpression cell line. The experimental procedure was identical to the inset in C. E) Immunofluorescence microscopy images of cells not expressing TbmtHMG44-HA (-tet) and after 24 hours of TbmtHMG44-HA expression (+tet d1). F) Immunofluorescence images of TbmtHMG44-ΔHMG-HA cells before construct expression (-tet) and after 24 hours of TbmtHMG44-ΔHMG-HA expression (+tet d1). The experimental settings were identical to E. Scale bars 5μm. G) Digitonin fractionations separating a mitochondrially enriched fraction of the cell from the rest of the cell body. Whole cells, the cytosolic fraction (soup) and the mitochondrially enriched fraction (pellet) were resolved on SDS-PAGE and visualized by western blotting. TbmtHMG44-ΔHMG-HA was detected with an anti-HA antibody. ATOM40 (a protein of the outer mitochondrial membrane) was traced as a control to demonstrate the mitochondrial enrichment in the pellet fraction. EF1α (a cytosolic protein) was used as a control for cytosolic enrichment.

To test the effects of TbmtHMG44 overexpression in PCF *T*. *brucei*, we generated cell line expressing an ectopic version of the protein (expressed construct shown in Fig 5A). We monitored growth rate of the TbmtHMG44-HA cell line after induction of expression over the course of 10 days and did not observe any growth defect (Fig 5C). Using the C-terminal HA epitope tag on the ectopic TbmtHMG44 we localized the protein in widefield immunofluorescence microscopy (Fig 5E). The signal co-localized with TAC102 and the DAPI signal of the kDNA. This agrees with our observations for *in situ* tagged TbmtHMG44 (Fig 1).

To assess the function of the HMG-box domain of TbmtHMG44, we then generated a second ectopic expression cell line. Instead of the full length TbmtHMG44 however, we inserted a truncated version of the gene (TbmtHMG44-ΔHMG). The construct used for this purpose is lacking the putative HMG-box domain (AA 22–80) of the protein (construct in Fig 5B). The ectopic expression of the TbmtHMG44-ΔHMG does not influence the growth rate of the cells (Fig 5D). When we performed widefield fluorescence microscopy of the cell line expressing TbmtHMG44-ΔHMG, we observed that, while some cells show a signal enrichment at the kDNA, in a large fraction of cells the signal is distributed throughout the cell body including non-specifically in the mitochondrion (Fig 5F). Biochemical fraction of digitonin solubilized cells showed an enrichment of the epitope tagged mutant version in the mitochondrial fraction, while some part of the protein was retained in the cytoplasmic fraction. Close inspection of the bands showed that the cytoplasmic fraction of TbmtHMG44-ΔHMG seemed larger than the fraction in the mitochondrion, hinting towards a lack of processing of the not imported fraction (Fig 5G).

### Combination of RNAi and proteomics reveals the organization of the kDNA-TAC interface

**Fig 6:**
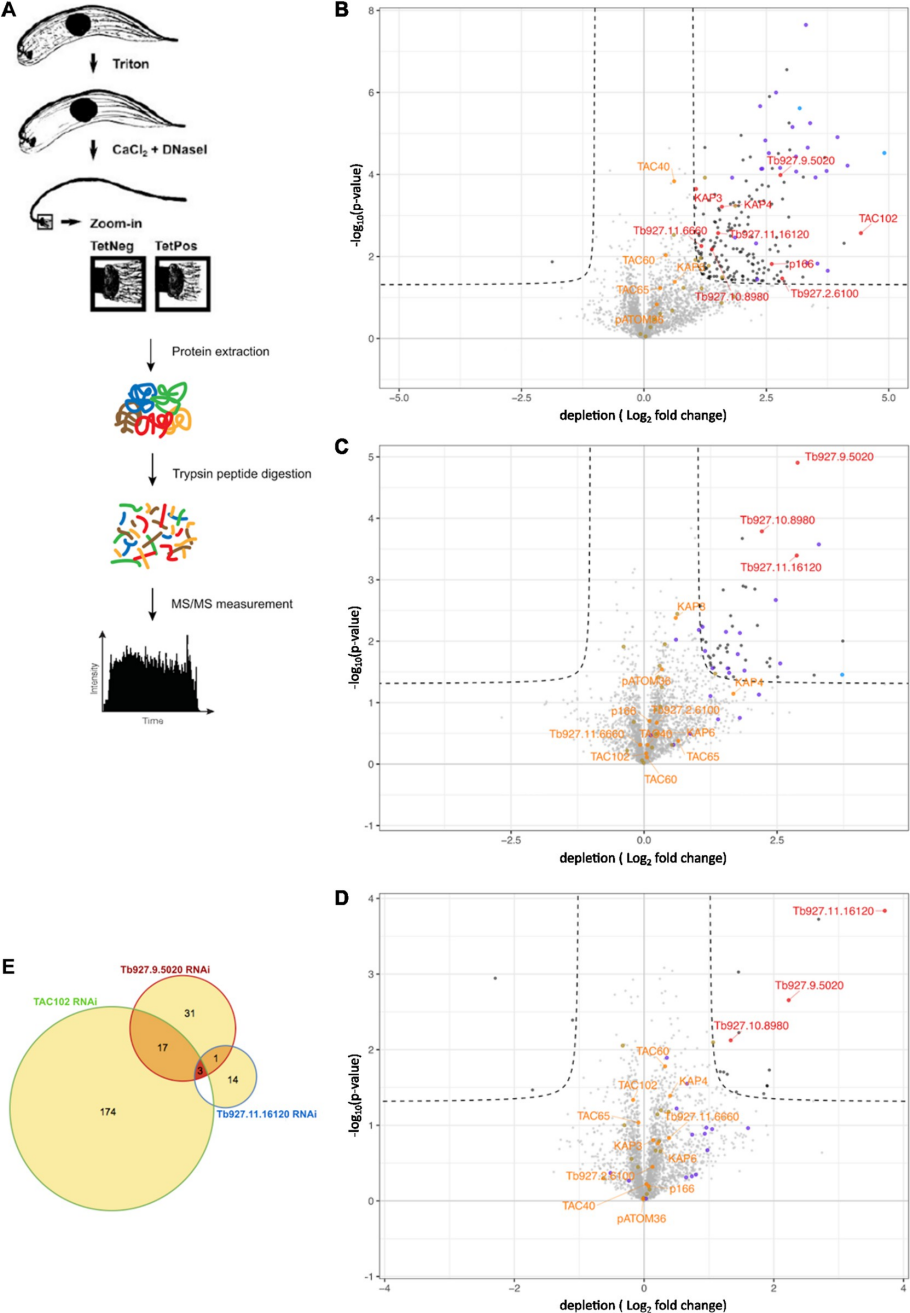
Combination of RNAi and proteomics targeting TAC102, TbmtHMG44 and TbKAP68 in BSF cells. A) Schematic overview of flagellar extraction from wild type cells and cells with TAC102, TbmtHMG44 or TbKAP68 RNAi in combination with quantitative mass spectrometry. B) Volcano plot showing proteins depleted in flagella extracted from cells after three days of TAC102 RNAi versus flagella extracts from wild type cells. The threshold was set as follows: p-value < 0.05 and log2FoldChange > 1 or <-1. C) Volcano plot as in B) from TbmtHMG44 RNAi cells at day three post induction. D) Volcano plot as in B) from TbKAP68 RNAi at day three post induction. E) Venn diagram of the proteins enriched in each of the experiments. Highlighted in blue: oxidative phosphorylation factors. Highlighted in purple: mitochondrial gene expression factors. Highlighted in khaki: kDNA replication factors. (additional information to these experiments in S7, S8, S9 and S10 Figs and S2 Table).

In order to characterize further interacting partners of TbmtHMG44 and TbKAP68 we developed a proteomics strategy that combines RNAi of a TAC protein or TAC associated component with the isolation of DNase treated flagella followed by quantitative mass spectrometry (Fig 6). From previous experiments, we know that TAC proteins remain associated at the kDNA proximal end of isolated flagella [22,24]. Thus, we expect that the closer the RNAi target is to the kDNA, the fewer proteins will be affected by the depletion of the target. This approach additionally allows us to further test our current model of the hierarchical organization [13,15]. We selected three proteins, TAC102, TbmtHMG44, and TbKAP68 to be targeted by RNAi. Specific BSF cells that are able to grow without kDNA, expressing one of these RNAi constructs were used in flagellar extractions with DNase treatment followed by mass spectrometry (experimental overview in Fig 6A, for details on these BSF cells see [24,36]). DNase treatment is required for a reliable sample preparation. The proteins that we identified in four replicates for the TAC102, the TbmtHMG44, or the TbKAP68 RNAi experiment are shown in volcano plots (thresholds: p-value <0.05 and a fold change -1< log_2_ >1) (Fig 6B–6D). Overall, the number of proteins identified by mass spectrometry is similar in all three experiments. However, while 194 proteins depend on the presence of TAC102, this number is lower for TbmtHMG44 (51) and lowest for TbKAP68, where only 15 interactors seem to depend on the presence of this protein (S7, S8 and S9 Figs). The only proteins that are depleted in all three experiments are TbmtHMG44, the hypothetical protein Tb927.10.8980 and TbKAP68 (Fig 6B–6E). Out of the 194 proteins that are affected by the depletion of TAC102, >75% are predicted to be localized in the mitochondrial organelle (S10A Fig). In the corresponding experiments targeting TbmtHMG44 and TbKAP68 the fraction of proteins with predicted mitochondrial localization is around 60% and 30%, respectively (S10B and S10C Fig). Neither the depletion of TAC102 nor of TbmtHMG44 or TbKAP68 changes the abundance of most characterized TAC components found in the flagellar extract (Fig 6B–6D). One exception is the inner mitochondrial membrane protein p166 that is decreased in abundance upon TAC102 RNAi (Fig 6B). This confirms the previously observed interaction of TAC102 and p166 [15]. Furthermore, the abundance of seven kDNA associated proteins KAP3, KAP4, Tb927.2.6100, Tb927.11.6660, TbmtHMG44, Tb927.10.8980 and TbKAP68 is affected by TAC102 depletion (Fig 6B). Tb927.2.6100 was previously described as a kDNA associated factor important for kDNA maintenance while Tb927.11.6660 was recently identified as an interactor of TAC102 [25,37]. Mass spectrometry also reveals the presence of 19 characterized mitochondrial DNA replication factors in the DNase treated flagellar extracts (S2 Table). They cover all stages of the replication process including minicircle release (TbTOPO2), replication initiation (UMSBP1/2), DNA synthesis (POLID, Primase 1 and Primase 2), as well as reattachment and gap closure (Pol β, Pol β PAK, MiRF172). Primase 2 was decreased b in abundance in all three proteomics experiments, while Pol β PAK, POLID, Primase 1, the kDNA associated proteins KAP3 and KAP4, the hypothetical protein Tb927.2.6100, the helicase TbPIF5 and the protease HslU2, were affected only by TAC102 knockdown. Thus 33% of the detected replication factors seem to depend on the presence of TAC102, while only Primase 2 (required for minicircle replication initiation) depends on all three proteins (TAC102, TbmtHMG44, TbKAP68).

## Discussion

A number of details about the TAC and its individual components have been elucidated since its discovery in 2003. However, how the TAC connects to the mitochondrial DNA remains unknown. Here we identify two novel components that are not part of the TAC and are required for kDNA maintenance. The combined RNAi/proteomics approach confirmed our current model of the hierarchical organization of the TAC, which correctly predicts that none of the known TAC components except p166 are depleted upon TAC102 RNAi (Fig 6). As described beforehttps://paperpile.com/c/6DBEWT/Wecl, the decrease in abundance of p166 during TAC102 RNAi is likely due to the direct interaction of the two proteins which has recently been described [15,38]. The proteomics data further indicate that a significant number of kDNA replication factors co-fractionate with isolated, DNase treated flagella. This supports the idea that the mitochondrial genome segregation and replication machinery are linked [39]. The replication proteins identified, include the essential replicative mini- and maxicircle polymerases POLIB (localized to the KFZ), POLIC (localized to the KFZ and the APS) and POLID (dynamic localization between KFZ and APS) (see S2 Table) [40–42]. The repair polymerase POLIA, which mostly localizes to the mitochondrial matrix, was not detected. Surprisingly, we also found several proteins that have been described to localize inside the kDNA disc, including the gap repair polymerase Pol β-PAK and the HMG-box containing proteins KAP3, KAP4 and KAP6 that are thought to fulfill the role of histone proteins in the kDNA [28,29,43]. This could either be a result of incomplete digest of the kDNA network or point towards a protein based structural element that connects these proteins through the TAC to the flagellum. Furthermore, the data suggests a possible link between the antipodal sites and the TAC. In the TAC102 depletion experiment we detected the following proteins that are known to exclusively localize to the antipodal sites: two topoisomerases (TbTOPOIA and TbTOPO2), two primases (PRI1 and PRI2), the single strand endonuclease (SSE1), p38, p93, the PIF5 helicase as well as the gap filling polymerase POL beta and the replication factor MiRF172. As mentioned before, we cannot exclude that some kDNA, which was protected from nucleolytic cleavage, remained in the proteomics samples and retained proteins attached to it. However, we consider it unlikely since in that case we should have seen significant differences between the wild type and RNAi induced samples from all three experiments; after all, three days post induction of RNAi targeting any of the three proteins, the vast majority of the cells had strongly reduced amounts of kDNA even before DNase I treatment (see Fig 2 and [24]). Overall, the proteomics data suggests that the kDNA replication machinery is assembled in several different compartments around the kDNA. Some but not all components of the replication machinery depend on the presence of the TAC and several of them seem not to depend on the presence of the kDNA to retain their localization.

The localization of TbmtHMG44 and TbKAP68 between TAC102 and the kDNA, as well as their distribution pattern during kDNA replication, is consistent with that of a TAC component (Fig 1) [15]. However, although the functional studies by RNAi show that both proteins are required for kDNA maintenance, their depletion does not lead to the characteristic missegregation phenotype seen for other TAC components (Fig 2) [13]. Furthermore, different from a typical TAC component the localization of TbmtHMG44 and TbKAP68 depends on the presence of kDNA prior to assembly of the proteins at the TAC-kDNA interface (Fig 1) suggesting a more direct interaction with the mitochondrial genome. Interestingly, once TbmtHMG44 and TbKAP68 are assembled at the TAC-kDNA interface, the kDNA is no longer required and can be removed by DNase I treatment, without affecting the association of these proteins with isolated flagella (S3 Fig). We analyzed the function of both proteins *in vivo* and *in vitro* and could show that they are required for maintenance of the kDNA and that TbKAP68 binds to DNA. Depletion of TbKAP68 leads to a rapid loss of mini- and maxicircles (Fig 3). Interestingly, the depletion of either of these two proteins leads to an initial increase of mainly maxicircle content (Fig 3A and 3B). This is a phenomenon that has also been observed during depletion of TOPIAmt, an enzyme responsible for processing minicircle replication intermediates [44]. One could speculate that the loss of the minicircle specific primase PRI2, together with some of the other factors depleted during TbmtHMG44 and TbKAP68 RNAi allow parts of the replication machinery to be redirected to the maxicircles, thus increasing the replication of this DNA species. An alternative explanation for the increase in maxicircle content might be an increased accessibility. As maxicircles are believed to be replicated within the network, they may profit from an overall less tight system that a weakened interface between the TAC and the kDNA might provide [45]. After this initial increase, also the maxicircles do not tolerate the knockdown of TbmtHMG44 or TbKAP68, as their levels decrease within a few days after RNAi induction.

Analysis of the minicircle replication intermediates reveals that while the overall levels of both replication intermediates decreases upon knockdown of either protein, we do see an increase of free replicated minicircles relative to the total amount of free minicircles detected in the population for TbKAP68 RNAi (Fig 3E). In case of TbmtHMG44 RNAi, the relative levels of replicated and non-replicated free minicircles remains stable over the course of RNAi induction (Fig 3F). This suggests that the replication still works to some degree in the absence of TbmtHMG44 or TbKAP68, and neither of the proteins is likely to be directly involved in kDNA replication. Without the use of nucleotide incorporation analyses, however, a direct role in kDNA replication cannot be excluded.

Recombinant TbKAP68 but not TbmtHMG44 is able to bind to DNA *in vitro*, however since both the minicircle and the control DNA were shifted there seems to be no obvious sequence specificity (Fig 4). The lack of DNA binding specificity might be due to missing posttranslational modifications or missing interactors in the *in vitro* setting. Alternatively, non-specific binding to the kDNA may be the true mode of interaction of TbKAP68.

Using transmission electron microscopy in combination with differential staining techniques, the ULF region of the TAC was previously divided into the inner and outer ULF [30]. The outer ULF consist of acidic proteins, while the inner region contains DNA and mostly basic proteins. These findings align well with newer findings on the ULF region. We now know that the acidic TAC/ULF protein p166 is associated with the inner mitochondrial membrane and directly interacts with the basic more kDNA proximal ULF protein TAC102 [22–24,37]. Towards the kDNA TAC102 is assumed to be connected to several proteins, including the recently characterized basic protein TAP110 (pI 8), which localizes closer to the kDNA than TAC102 [25]. Since we have no evidence that TAC102 directly binds to the kDNA and based on recent ultrastructure expansion microscopy (U-ExM) data that shows a clear gap between TAC102 and the kDNA, we suggest that there are additional components required to connect the TAC and the kDNA [25]. Two of these components are the very basic proteins TbKAP68 and TbmtHMG44 that localize between TAC102 and the kDNA. They are distinct from currently described TAC components as they (i) require the presence of the TAC and the kDNA for their localization and (ii) upon RNAi depletion lead to a kDNA loss but not missegregation phenotype. Based on these findings we suggest that TbKAP68 and TbmtHMG44 are part of a novel complex that connects the TAC to the kDNA. For TbKAP68 we could demonstrate DNA binding *in vitro*.

## Materials and methods

### *T*. *brucei* cell culture conditions

Monomorphic New York single marker (NYsm) bloodstream form (BSF) *T*. *brucei* cells [46] and single marker γL262P cells [36] were cultured in Hirumi-modified Iscove’s medium 9 (HMI-9) supplemented with 10% fetal calf serum (FCS) [47] and 2.5 μg/ml, geneticin at 37°C and 5% CO_2_. 29–13 double marker procyclic form *T*. *brucei* cells [46] were cultured in semi-defined medium-79 (SDM-79) supplemented with 10% FCS, 15 μg/ml geneticin and 25 μg/ml hygromycin at 27°C. For the analysis of the RNAi phenotype, cells containing RNAi constructs, were grown with 1 μg/ml tetracycline or without tetracycline and kept in the exponential phase. For the growth curves we measured the cell density by using a Neubauer chamber to count cell density every 24h. The BSF single marker and PCF double marker cell lines were obtained from the established collection of the Institute of Cell Biology, University of Bern, Bern, Switzerland. The single marker γL262P cell line is a kind gift of Achim Schnaufer. The NYsm TAC102 RNAi and the single marker γL262P p197 RNAi cell lines were obtained from earlier studies [15,24]. Depending on the cell line, 2.5 μg/ml geneticin, 0.5 μg/ml puromycin, 2.5 μg/ml phleomycin, 5 μg/ml blasticidin or 2.5 μg/ml hygromycin was added to the media. For inducing RNAi 1 μg/ml tetracycline was used.

### YFP-TAC102 immunoprecipitation (IP) and mass spectrometry analysis of the IP

TAC102 was N-terminally YFP-tagged in SmOxP927 cells by using the pPOTv4 vector as previously described [48]. We used the following primers for the tagging: FWD 5’-AAAGAGTGAGTGAGG- TGAGAGCGAAGAATTGCGGACAGCGCACTTCATACTCTGATCTTTCCCTTTACCCTAGCGACAAAGTATAATGCAGACCTGCTGC-3’, REV 5’-TGAGAGCCAGAGTGGTCAGCCTTCCTTGAAGCAGCGGATTCCTTCCGATCCTGCTTAGCGCCGCACGAGGCCGATACATACTACCCG-ATCCTGATCC-3’. The transfection was achieved by electroporation (1.3kV for 100 μs). The cell preparation and mass spectrometry were performed as previously described [49]. In brief: 10^10^ cells were cytoskeleton extracted and sonicated (3×10 sec at 10 microns amplitude) to break cellular microtubules, followed by a 30 min incubation with PEME, 0.2 M NaCl and Protease Inhibitors at 4°C. After centrifugation, the pellet was resuspended in 0.05% Nonidet P-40 in PBS, and was further fragmented by sonication (6×20 sec at 10 microns amplitude) and added to dynabeads (ThermoFisher Scientific) crosslinked with the α-GFP antibody (Roche). Beads were incubated with 50 mM Tris pH 7.5, 0.3% SDS and 1 mM EDTA to elute the bound material. The eluted sample was then fractionated by SDS-polyacrylamide gel electrophoresis and visualized by Sypro Ruby. Each lane was cut into small pieces and send to mass spectrometry, omitting the tubulin band. The rest was analyzed with the Q Exactive mass spectrometer. MS/MS spectra were searched against a database based on *T*. *brucei* genome version 9.0 using the Central Proteomics Facility Pipeline version 2.1 of Sir William Dunn School of Pathology, University of Oxford, Oxford, United Kingdom. The enrichment of the proteins was calculated by the spectral index ratio of the eluate to the flow-through. The abundance represents the spectral index ratio of the eluate to the median of the eluate.

### Flagellar extraction for fluorescence microscopy and mass spectrometry analysis

We used five million cells for flagellar extraction for fluorescence microscopy and 20 million cells for flagellar extraction for mass spectrometry analysis. The cell culture was supplemented with EDTA (pH 8.0) to a final concentration of 5 mM prior to centrifugation at 2500 rcf for 8 min. The pellet of cells was then washed with 1 ml of extraction buffer basic (10 mM NaH_2_PO_4_, 150 mM NaCl, 1 mM MgCl_2_ at pH 7.2). Then cells were resuspended in extraction buffer I (extraction buffer basic containing 0.5% Triton X-100; resuspension ratio: 5 million / 20 μl) and extracted for 10 min on ice. The extracted cells were then collected by centrifugation at 3000 rcf for 3 min at 4°C and washed with extraction buffer basic. For depolymerization of subpellicular microtubules the extracted cells were resuspended in extraction buffer II (extraction buffer basic containing 1 mM CaCl_2_; resuspension ratio: 5 million / 30 μl) and incubated for 45 min on ice. For DNase I treatment the extraction buffer II was supplemented with DNase I (Roche) to a final concentration of 100 μg/ml prior to resuspension. The flagella were then collected by centrifugation at 3000 rcf for 3 min at 4°C and washed twice with PBS. All extraction buffers used for the isolation of flagella for mass spectrometry were supplemented with 2x concentrated cOmplete protease inhibitor cocktail (Roche). TbKAP68 flagellar extraction for immunofluorescence microscopy was performed as described above. For the TbmtHMG44 flagellar extraction, cells were cytoskeleton extracted with 1% Nonidet P-40 in 100 mM PIPES pH 6.9, 1 mM MgSO_4,_ 100 mM EDTA and 2 mM EGTA for 5 min at room temperature. The cytoskeletons were separated from soluble material by centrifugation and then the cytoskeleton pellet was resuspended in 20 mM PIPES pH 6.9 containing 65 mM CaCl_2_ and incubated for 25 min on ice. For 10 min, the flagella were then treated with DNase buffer (New England Biolabs) that was either supplemented with DNase I, or used plain (at room temperature). After an additional centrifugation step the pellet was distributed on microscopic slides and the immunofluorescence staining was performed as described further down in this chapter.

### Mass spectrometry and data analysis of flagella

Flagella were extracted as described above. We used flagella from TAC102 RNAi, TbmtHMG44 RNAi and TbKAP68 RNAi cell lines, either non-induced or induced for three days. The isolated flagella were resuspended in LDS sample buffer (Invitrogen, NU PAGE) and proteins were denatured at 70°C for 10 minutes. The sample preparation and mass spectrometry were performed as previously described [25]. In brief: The protein lysates were each separated on 10% gradient SDS gels (ThermoFisher Scientific) for 8 min at 180 V. Then the proteins were fixed and stained with a Coomassie solution, and the gel lane was cut into slices, minced, and destained. Proteins were reduced in 10 mM DTT for 1h at 56°C and then alkylated with 50 mM iodoacetamide for 45 min, at room temperature, in the dark. To obtain peptides, the proteins were digested with trypsin overnight at 37°C and the peptides were extracted from the gel using acetonitrile and ammonium bicarbonate [49]. For mass spectrometric analysis, peptides were separated on a 50 cm self-packed column (New Objective) with 75 μm inner diameter filled with ReproSil-Pur 120 C18-AQ (Dr. Maisch GmbH) mounted to an Easy-nLC 1200 (ThermoFisher Scientific) and sprayed online into an Orbitrap Exploris 480 mass spectrometer (ThermoFisher Scientific). We used a 103-min gradient from 3% to 40% acetonitrile with 0.1% formic acid at a flow of 250 nL/min. The mass spectrometer was operated with a top 20 MS/MS data-dependent acquisition scheme per MS full scan. Mass spectrometry raw data were searched using the Andromeda search engine integrated into MaxQuant software suite 1.6.5.0 using the TriTrypDB-46_TbruceiTREU927_AnnotatedProteins protein database (11,203 entries) [50,51,52]. For the analysis, carbamidomethylation at cysteine was set as fixed modification, while methionine oxidation and protein N-acetylation were considered as variable modifications. Match between run option was activated.

### Bioinformatics analysis

Contaminants, reverse database hits, protein groups only identified by site, and proteingroups with none unique and less than 2 peptides were removed by filtering from the MaxQuant proteinGroups file. Missing values were put in by shifting a beta distribution obtained from the LFQ intensity values to the limit of quantitation. Further analysis and graphical representation was performed in the R framework incorporating ggplot2 package in-house R scripts [53,54].

### Extraction of DNA from NYsm and γL262P cells for PCR and Southern blot analysis

Total DNA was isolated from mid-log phase cells. For this, the 50 million cells were centrifuged for 8 min at 2500 rcf, washed with NTE buffer and then resuspended in 0.5 ml NTE buffer. Cells were lysed by addition of 25 μl of 10% SDS. RNA was degraded by addition of 5 μl RNase A (20 mg/ml, Sigma-Aldrich) and incubation for 1 h at 37°C. The proteins we degraded by addition of 25 μl proteinase K (10 mg/ml) and further incubation for 2 h at 37°C. For the isolation of DNA 0.5 ml phenol was added to the reaction and the mixture was well mixed. Separation of the aqueous and organic phase was performed by centrifugation for 8 min at 16000 rcf. The aqueous phase was transferred to a new tube and an equal amount of chloroform was added. The mixture was well mixed. The centrifugation was repeated, and the upper phase was transferred to a new tube. For precipitation of the DNA we added 1/10 volume of 3 M Na-acetate (pH 5.2) and 2x the volume of absolute ethanol. Then we incubated the DNA for 1 h at -80° C or overnight at -20°C. The precipitated DNA was collected by centrifugation at 16000 rcf for 30 min at 4°C. The pellet was then washed with 1 ml of 70% ethanol and the centrifugation was repeated. After the wash we dried the DNA at 55°C and then resuspended in 50–100 μl of sterile MilliQ water.

### Cloning of the tagging and RNAi constructs

For the *in situ* tagging of TbKAP68 at the C-terminus we used a PTP tag that was integrated into a pLEW100 based plasmid [55]. We obtained the tagging construct by amplification of the TbKAP68 gene ORF positions 1330 to 1806 from genomic NYsm DNA by PCR. For this, we used primers that contained adaptor sequences with the respective restriction sites (FWD 5’-gtacGGGCCCttgtctagtcccatttgggtgactcc-3’, REV 5’-gtacCGGCCGgagtgtggtgccctggggtcttgtg-3’). The amplified ORF region was then cloned into the plasmid by using the ApaI and EagI sites of the plasmid. The resulting tagging plasmid we then linearized with AatII prior to transfection. For the C-terminal HA-tagging of TbmtHMG44 we used the pMOTag4H vector [56]. We utilized following primers: FWD 5’-TGATGTTCTGGAGCGGACGGGCTGTTTCCGCAGCAAAGAAGCTAACCAGCTGCTT-AGGGAGACGTACATAAACCCTACAAGCAAGAAAAAAGGGAAAGAAGGTaccGGGcccCCCctcGAG-3’ and RV 5’-AAGTAACATAATGCAAC-AAGAAAAGGAGGAAAACATAAAAAGTAATCATGAGAGAGGGAAAAAATGAGAGGAAATGGTTTATGTATCTATAATCGTTACTTGGCGGCCGCTCTAGAACTAGTGGAT-3’. For the cloning of the TbKAP68 RNAi construct we used the ORF position 467 to 966 of the TbKAP68 gene as target. We amplified this ORF region as described above using the following primers: FWD 5’-gtaaGGATCCAAGCTTaggtaaaccggaaggacgtt-3’, REV 5’-cttaCTCGAGTCTAGAatccctgacgttgacgaagt-3’. The TbmtHMG44 RNAi was targeted against the ORF (285 bp– 797 bp; FWD 5’-CTTAAAGCTTGGATCCAGTAACGATTATA-GATACATAAACC-3’ and RV 5’-CTTATCTAGACTCGAG CCGTCACAATCTGCTTCTAC-3’) or against the 3’UTR (1187 bp– 1524bp; FWD 5’-AGTCGAAGCTTGGATCCACACCGAAAAGGCATTCAAC-3’ and RV 5’-TCCGATCTAGACTCGAGACTGGGCAAATAG-CCGTATG-3’). The amplified construct was then inserted twice into a tetracycline (tet) inducible RNAi vector containing a stuffer sequence (modified pLew100-expression vector) to generate the hairpin-loop containing dsRNA coding sequence [57]. The restriction sites BamHI, HindIII, XbaI and XhoI were used to do so. The final plasmid we then linearized with NotI prior to transfection. All restriction enzymes we used, were bought from New England Biolabs. The cloning of the TbmtHMG44 (FWD 5’-gatcAAGCTTatgaggcggtgctgttgtg-ccaaaagc-3’, REV 5’-gttaCTCGAGttctttcccttttttcttgcttgtagggtttatgtac-3’) and TbmtHMG44-ΔHMG (FWD 5’-gatcAAGCTTatgaggcggtgctgttgtgccaa-aagcggaaggcctcagtttctcattgacagtccgcacgttggggccatgagagtgccgaac-3’, REV: same as above) overexpression constructs was performed as described previously using the primers indicated in brackets [25].

### Transfections

To obtain transgenic cell lines, we transfected cells with the constructs described above. We integrated the constructs by making use of the homologous recombination mechanism of the cells. The transfection mixtures were prepared as follows: 10 μg of linearized plasmid were mixed with 3x and 1x transfection buffer (90 mM Na- phosphate (pH 7.3), 5 mM KCl, 0.15 mM CaCl_2_, 50 mM HEPES (pH 7.3)) to a total volume of 110 μl 1x concentrated transfection buffer [58]. BSF transfection was performed with 40 million cells and PCF transfection was performed with 100 million cells. Cells were collected by a centrifugation at 2500 rcf for 8 min, mixed carefully with the transfection mixture and transferred to an Amaxa Nucleofector cuvette. The program X-001 or Z-001 of the Amaxa Nucleofector II was used for BSF transfection and the program X-014 for PCF transfections [59]. After transfection the cells were recovered in the respective medium for 20h. After the recovery we then added the respective antibiotics to select for integration of the transfected constructs. For selection of the PTP *in situ* tagging of TbKAP68 and all RNAi cell lines (BSF) we used 5 μg/ml blasticidin. For selection of the *in situ* tagging of TbmtHMG44 with HA (BSF) we used 2.5 μg/ml of hygromycin. For selection of TbmtHMG44-HA and TbmtHMG44-ΔHMG-HA overexpressors, we used 1 μg/ml puromycin.

### Quantitative Real-Time PCR

The extraction of total RNA was performed with RiboZol (Ameresco). 5 μg of RNA was treated with 0.5 μl DNase I (New England Biolabs) for 15 min at 37°C. Purification was performed with 200 mM NaOAc pH 4, phenol, chloroform/isoamylalcohol. To synthesize cDNA the Omniscript reverse transcription kit (Qiagen) was used and performed as described in the manual. 1 μg of the template and random hexamers as primers were incubated for 1 h at 37°C, followed by heat inactivation for 5 min at 93°C. The MESA Green qPCR MasterMix Plus for SYBR assay (Eurogentec) was used to perform the quantitative Real-Time PCR (qPCR) and the cDNA was mixed as described in the manual. The qPCR was performed with the ABI Prism 7000 Sequence Detection System (Applied Biosystems) and the data were analyzed by using the 7000 System SDS software v1.2 (Applied Biosystems). For TbmtHMG44 we used following primer sequences: FWD 5’-ACCAGCTGCTTAGGGAGACG-3’, RV 5’-GAACACCAGCACTCACCCGT-3’. Normalization of the CT-values was performed to the housekeeping gene α-tubulin (FWD 5’-CGCTATTATTAGAACAGTTTCTGTAC-3’, RV 5’-GTTACCAACCTGGCAACCA-3’) and the uninduced value equals one.

### Northern blot analysis for detection of RNA

For the isolation of RNA, we collected cells, 50 million of each, non-induced NYsm TbKAP68 RNAi cells and cells at day three post induction of the RNAi by centrifugation at 2500 rcf for 8 min. The pellet was washed with 1 ml PBS and then cells were lysed by resuspension in 1 ml of TRIZOL (Ambion). 0.2 ml chloroform was added to the lysed cells and the mixture was vortexed for approximately 15 s prior to centrifugation at 1100 rpm for 10 min at 4°C. The aqueous phase was then transferred to a fresh tube and mixed with the equal volume of isopropanol. Again, the mixture was vortexed for around 15 s prior to centrifugation as described above. The precipitated RNA was then washed with 1 ml of 70% ethanol (same centrifugation as described above). The isolated RNA was then dried at 55°C and resuspended in 50 to 100 μl nuclease free water. For the northern blot analysis, 10 μg of RNA/sample were used to perform gel electrophoresis. For this we mixed the RNA with sample preparation buffer (0.1 mg/ml ethidium bromide, 15% formaldehyde, 0.1 M MOPS, 0.3 M Na-acetate, 0.05 M EDTA (pH 8), bromophenol blue) and incubated for 15 min at 65°C. After cooling down the sample to room temperature, the samples were loaded onto a 1.4% agarose gel and RNA was resolved at 100V for approximately 2 h. As a running buffer we used MOPS (pH 7) containing 5.92% formaldehyde. After the electrophoresis the RNA was transferred to a Hybond nylon membrane by capillary transfer with 20x SSC (3 M NaCl, 0.3 M Na-citrate, pH 7) over night. After the transfer the membrane was auto-crosslinked with the Stratagene UV-Stratalinker. For detection of the TbKAP68 mRNA, membranes were first pre-wet in 5xSSC and then blocked in hybridization solution (5x SSC, 1:12.5 100x Denhardt’s (2% BSA, 2% polyvinylpyrrolidone, 2% Ficoll), 50 mM NaHPO_4_ (pH 6.8), 1% SDS, 100 μg/ml salmon sperm DNA) at 65°C for one hour. To generate the probe, we used the same PCR product we also used for cloning of the RNAi construct. In a first step, the DNA was denatured at 95°C for 5 min. Then we followed the manufacturer’s protocol for radioactive labelling through Klenow (Random primed DNA labeling kit, Roche). The reaction was stopped by the addition of 0.2 M EDTA (pH 8.0) and added to the membrane in hybridization solution. The membrane was hybridized overnight at 65°C. The next day membrane was washed twice with 2x SSC, 0.1% SDS and twice with 0.2x SSC, 0.1% SDS at 60°C. Then the membrane was exposed to a storage phosphor screen (Amersham Bioscience) for around 24h and scanned by a Storm PhosphoImager (Amersham Bioscience). For normalization, the membrane was blocked as described above and then probed for the 18S RNA. The 18S rRNA probe was generate as follows: 1.8 μl 18S rRNA (10 μM) was mixed with 12.5 μl water, 2.7 μl γ-32P-ATP (1 MBq), 2 μl PNK buffer (10x) and 1 μl T4 PNK and incubated for 30 min at 37°C. The reaction was stopped with 5 μl EDTA (0.2 M) and 75 μl TE buffer and incubated 5 min at 98° C. The probe was then quenched for 2 min on ice and 50 μl of the reaction was added to the membrane in hybridization solution. Hybridization and washes were performed as described above. Exposure time was 15 min and the screen was scanned as described above.

### Immunolabeling for microscopy

Approximately one million cells were collected by centrifugation for 3 min at 1800 rcf. Cells were washed with 1 ml PBS and then resuspended in 20 μl PBS and spread on a glass slide for regular epifluorescence microscopy or on a plasma coated Nr. 1.5 cover glasses (Marienfeld) for 2D-STED microscopy. After the cells had settled, we fixed them for 4 min with 4% PFA (in PBS). After fixation we permeabilized with 0.2% Triton-X 100 (in PBS, 5 min). For immunolabeling of flagellar extracts, we resuspended 5 million flagella as described above and fixed them as described above. Cells and flagella were blocked with 4% BSA in PBS for 30 min. The primary and secondary antibodies were incubated for 45–60 min and diluted in blocking solution as follows: polyclonal rabbit-anti-Protein A (Sigma-Aldrich) detecting the PTP epitope 1:2000, rat YL1/2 antibody detecting tyrosinated tubulin as present in the basal body [60] 1:100000, monoclonal mouse TAC102 antibody [24] 1:2000, rabbit-anti-HA (Sigma-Aldrich) 1:1000, rabbit-anti-ATOM40 1:10000, Alexa Fluor 488 Goat-anti-Rabbit IgG (H+L) (Invitrogen), Alexa Fluor 594 Goat-anti-Mouse IgG (H+L) (Molecular probes), Alexa Fluor 488 Goat-anti-Mouse IgG (H+L) (Invitrogen), Alexa Fluor 647 Goat-anti-Rat IgG (H+L) (Life technologies), Alexa Fluor 594 Goat-anti-Rat IgG (H+L) (Invitrogen) all 1:700 or 1:1000. For 2D-STED microscopy we used antibodies with following dilutions: Polyclonal rabbit-anti-Protein A antibody (Sigma-Aldrich) and monoclonal mouse-anti-TAC102 antibody as described above and the secondaries Alexa Fluor 594 goat-anti-Rabbit IgG (H+L) (Invitrogen) and the Fluor Atto647N Goat-anti-Mouse IgG (H+L) (Molecular probes) we used 1:500 in 4% BSA and incubated for one hour on the cover slips. After each antibody incubation cells were washed 3x with 0.1% Tween-20 (in PBS). A final wash with PBS was performed prior to mounting. Cells were mounted with ProLong Gold Antifade Mounting Medium with DAPI (4’,6-diamidine-2-phenylindole) (Invitrogen). The YL1/2 antibody is a kind gift of Keith Gull. The ATOM40 antibody is a kind gift from André Schneider.

For super-resolution microscopy, cells were spread on a glow discharged cover glass (coverslip thickness no. 1.5, plasma coating for 30 s with FEMTO SCIENCE CUTE discharger) instead of a slide.

### Epifluorescence, confocal and 2D-STED

For regular epifluorescence microscopy images were acquired with the Leica DM5500 B microscope with a 100x oil immersion phase contrast objective and analyzed using LAS AF software (Leica) and ImageJ. For confocal and 2D-STED super-resolution microscopy we used the SP8 STED microscope (Leica, with a 100x oil immersion objective and the LAS X Leica software). Images were acquired as z-stacks with a z-step size of 120 nm and a X-Y resolution of 37.9 nm. We used the 594 nm excitation laser and the 770 nm depletion laser to acquire the TbmtHMG44 and TbKAP68 signal. The TAC102 signal we acquired with the 647 nm excitation laser and the 770 nm depletion laser. The DAPI signal was acquired with confocal settings. To deconvolute the pictures we used the Huygens professional software.

### Cytoskeleton extraction for fluorescence microscopy

For cytoskeleton extraction the cells were harvested, washed, resuspended and spread on a slide as described above. Then the cells were incubated for 5 min with extraction buffer (100 mM PIPES (pH 6.8), 1 mM MgCl_2_) containing 0.05% Nonidet P-40. After the extraction, the cells were washed twice with extraction buffer and the immunolabeling was performed as described above.

### SDS-PAGE and western blot analysis

Cell extracts were mixed with Laemmli buffer to a final concentration of 5 million cell-equivalents/15 μl. Either one million or five million cell-equivalents (depending on the experiment) were loaded per lane on an SDS-polyacrylamide gel. The gels were resolved by 80-120V and transferred (wet transfer, 100V for 1h) onto PVDF Immobilon- FL transfer membranes (0.45 μm, MILLIPORE), followed by blocking (5% milk in PBS + 0.1% Tween-20 (PBST)) for 1 h at room temperature. Primary antibodies were incubated for either 1 h at room temperature or overnight at 4°C. Secondary antibodies were applied for 1 h at room temperature. All used antibodies were diluted in blocking solution. The following antibodies were used: mouse anti-TAC102 (1:1000, [24]), rabbit anti-HA (1:1000, Sigma-Aldrich), rabbit anti-ATOM40 (1:10000, [61]), rabbit anti-mouse HRP-conjugated (1:10000, Dako) and swine anti-rabbit HRP-conjugate (1:10000, Dako), rabbit peroxidase anti-peroxidase soluble complex (PAP) (1:2000, for detection of the PTP tag). After antibody incubation, the membranes were washed three times with PBST. A final wash with PBS was performed prior to detection of the protein with the SuperSignal West Femto Maximum Sensitivity Substrate (ThermoFisher Scientific). The Amersham Imager 600 (GE Healthcare) was used to visualize the protein bands on the blot.

### Southern blot analysis for the detection of mini- and maxicircle DNA

For the mini- and maxicircles 5 μg of total DNA, digested with HindIII and XbaI, were loaded, while for the free minicircles 8–10 μg of total DNA was used. The experiment was performed as described previously [38]. For detection either the DIG high prime DNA labeling and detection kit (ROCHE) or α-^32^P-dCTP and/or α-^32^P-dATP were used. Minicircle primers: FWD 5’-TATGGGCGTGCAAAAATACA-3’, RV 5’-CGAAGTACCTCGGACCTCAA-3’. Maxicircle primers: FWD 5’-GCCTGCTGGGAATTGTTCTG-3’, RV 5’-GATCCACGTTCAATGCACCG-3’. Tubulin primers: FWD 5’-CTAACATACCCACATAAGACAG-3’, RV 5’-ACACGACTCAATCAAAGCC-3’.

### Purification of recombinant proteins

Encoded by the plasmid pET22b+ or pMBP-parallel1 respectively, C-terminally His-tagged TbKAP68 variants (rKAP68-His and rKAP68-ΔN-His) and N-terminally MBP-tagged TbmtHMG44 (MBP-rmtHMG44) were expressed in BL21 *Escherichia coli*. Expression was induced with 1 mM isopropyl-β-D-thigalactoside (IPTG) at a growth temperature of 28°C for 4 hours. To purify the recombinant proteins, an amylose resin column (New England Biolabs) or a Nickel loaded HisTrap HP column (GE Healthcare) were used. MBP-rmtHMG44 was purifed accoring to the manufacturer’s protocol and eluted with a buffer containing 20 mM Tris-HCl pH 7.4, 0.2 M NaCl, 1 mM EDTA, 10 mM maltose. For the MBP-cleavage of recombinant TbmtHMG44 the AcTEV Protease (Invitrogen) was added to the protein as suggested from the manual. The protein was then stored at 4°C.

For the purification of rKAP68-His and rKAP68-ΔN-His, the cells were harvested by centrifugation, and resuspended on ice in 16 ml 20 mM Tris, 4 mM MgCl_2_ (pH 8 at 4°C) per liter of culture volume. The cells were lysed using a Microfluidizer at 10000 PSI. The lysate was then treated with 500 units of Benzoase Nuclease (Sigma-Aldrich) per liter of culture volume, for 20 min on ice. Triton was added to a final concentration of 0.5% v/v, and the lysate was incubated for 10 min on ice. The lysate was then centrifuged at 3000 rcf for 10 min, and the pellet was resuspended in 83 ml/L 50 mM Tris, 100 mM NaCl, 1 mM EDTA, 10 mM DTT (pH 8 at 4°C), and incubated for 10 min on ice. Insoluble substances were then pelleted at 3000 rcf, for 10 min. The pellet was resuspended in 83 ml/L 50 mM Tris, 100 mM NaCl, 1 mM EDTA (pH 8 at 4°C), and incubated for another 10 min on ice. The sample was pelleted at 3000 rcf for 10 min. The pellet was resuspended in 16 ml/L 50 mM Tris, 100 mM NaCl (pH 8 at 4°C), and incubated for 10 min. Then it was centrifuged for another 10 min at 3000 rcf and the pelleted inclusion bodies were solubilized in 16 ml/L 8 M urea, 50 mM Tris, 400 mM NaCl (pH 8 at 4°C). Insoluble components were removed by high speed ultracentrifugation, and the recombinant protein was purified on a Nickel loaded HisTrap HP column (GE Healthcare) using the ÄKTA prime system. The protein was eluted in a gradient from 50 mM Tris, 500 mM NaCl to a buffer containing the same ingredients plus 500 mM imidazole, and eluted at about 20% of the gradient, with 100 mM Imidazole. The protein was then dialyzed into 50 mM Tris, 100 mM NaCl overnight, and stored at -20°C. Protein concentrations were measured using the NanoDrop 2000 spectrophotometer.

### Electro mobility shift assay (EMSA)

The LightShift Chemiluminescent EMSA assays were performed according to the manufacturer’s kit protocol, with small adaptations in the shift assay (ThermoFisher Scientific). We did not use dI∙dC and adapted the type and amounts of detergent as described further down in the text. We used 73 bp or the conserved minicircle sequence as DNA bait: FWD 5’-GAAAAAACGGAAAATCTTATGGGCGTGCAAAAATACACATACACAAATCCCGTGCTTATTTTAGGCCATTTTT-3’ and REV 5’-AAAAATGGCCTAAAATAAGCACGGGATTTGTGTATGTGTATTTTTGCACGCCCATAAGATTTTCCGTTTTTTC-3’. The binding reactions were performed with the kits binding buffer and supplemented with 0.1% digitonin, 0.01% BSA, 20 fmol biotinylated DNA, and 4 pmol unlabeled DNA. Variable amounts of recombinant protein were used. After resolving on a 6% acrylamide gel and transfer onto a nylon membrane, the biotinylated DNA was detected with streptavidin-HRP conjugate according to the manufacturers protocol (ThermoFisher Scientific). As a control the Epstein-Barr nuclear antigen protein extract and DNA were used, as provided with the kit.

### Affinity precipitation of rKAP68-His / rKAP68-ΔN-His with MBP-rmtHMG44

Same amounts of the recombinant proteins were mixed and imidazole was added to an end concentration of 10 mM. We either used rKAP68-His or rKAP68-ΔN-His, but in both cases mixed with MBP-rmtHMG44. For the control sample rKAP68-His was replaced with the same volume of water. After 1 h incubation at room temperature, the samples were mixed with HisPur Ni-NTA resin (ThermoFisher Scientific) that had been previously washed twice with equilibration buffer (PBS, 10 mM imidazole, pH 7.4). The samples were incubated for 45 min at room temperature on a rotor at 15 rpm. After a 2 min centrifugation step at 700 rcf the supernatant (= flowthrough) was replaced with wash buffer (PBS, 25 mM imidazole, pH 7.4) and the samples were washed five times. The samples were then incubated with elution buffer (PBS, 250 mM imidazole, pH 7.4) for 10 min at room temperature and centrifuged to harvest the elution fraction. MBP-rmtHMG44 was tracked throughout experimental procedure using western blot analysis with a commercially available anti-MBP antibody (New England Biolabs).

### Digitonin fractionation of TbmtHMG44-ΔHMG expressing cells

Prior to digitonin extraction cells were induced with tetracycline for 24h for expression of TbmtHMG44-ΔHMG. After 24 h cells were harvested and washed with PBS. To obtain crude mitochondria enriched fractions we incubated 50 million cells on ice for 10 min in SoTE (0.6 M sorbitol, 20 mM Tris-HCl pH 7.5, 2 mM EDTA pH 8) containing 0.015% (w/v) digitonin, which selectively solubilizes the plasma membrane only. We then centrifuged for 5 min at 6800 rcf at 4° C to obtain a cytosol-enriched supernatant and a mitochondria-enriched pellet. Equivalents of 2 million cells of each fraction, including whole cells, were analysed by SDS-PAGE and western blotting.

## Supporting information

S1 Fig YFP tagged TAC102 localizes near the kDNA throughout the cell cycle. (TIF)

S2 Fig Localization of TbKAP68 across the cell cycle. (TIF)

S3 Fig Analysis of the dependencies of TbmtHMG44 and TbKAP68 localizations on the TAC and the kDNA. (TIF)

S4 Fig Phenotype upon depletion of TbKAP68 or TbmtHMG44 in BSF cells. (TIF)

S5 Fig Southern blot analysis of TbKAP68 and TbmtHMG44 RNAi cells. (TIF)

S6 Fig Protein purifications, additional replicates and further experiments supporting the interaction data of TbKAP68 and TbmtHMG44. (TIF)

S7 Fig Proteins that significantly changed in expression levels in TAC102 and TbmtHMG44 RNAi. (TIF)

S8 Fig Heat maps of proteins candidates from mass spectrometry analysis of TAC102 and TbmtHMG44 RNAi. (TIF)

S9 Fig Proteins that depend on the presence of TbKAP68. (TIF)

S10 Fig Mitochondrial prediction for the proteins depleted upon A) TAC102, B) TbmtHMG44 or C) TbKAP68 RNAi. (TIF)

S1 Table Candidates of the YFP-TAC102 immunoprecipitation. (TIF)

S2 Table kDNA replication/segregation factors identified in the flagellar 112 extract mass spectrometry analysis. (TIF)

S1 Data Numerical basis of graphs shown in the manuscript. (XLSX)
