# Supplementary figures and images for: Characterization of two novel proteins involved in mitochondrial DNA anchoring in Trypanosoma brucei
Source: PLoS Pathog. 2023 Jul 17;19(7):e1011486. doi: 10.1371/journal.ppat.1011486 (PMC10374059; doi:10.1371/journal.ppat.1011486)

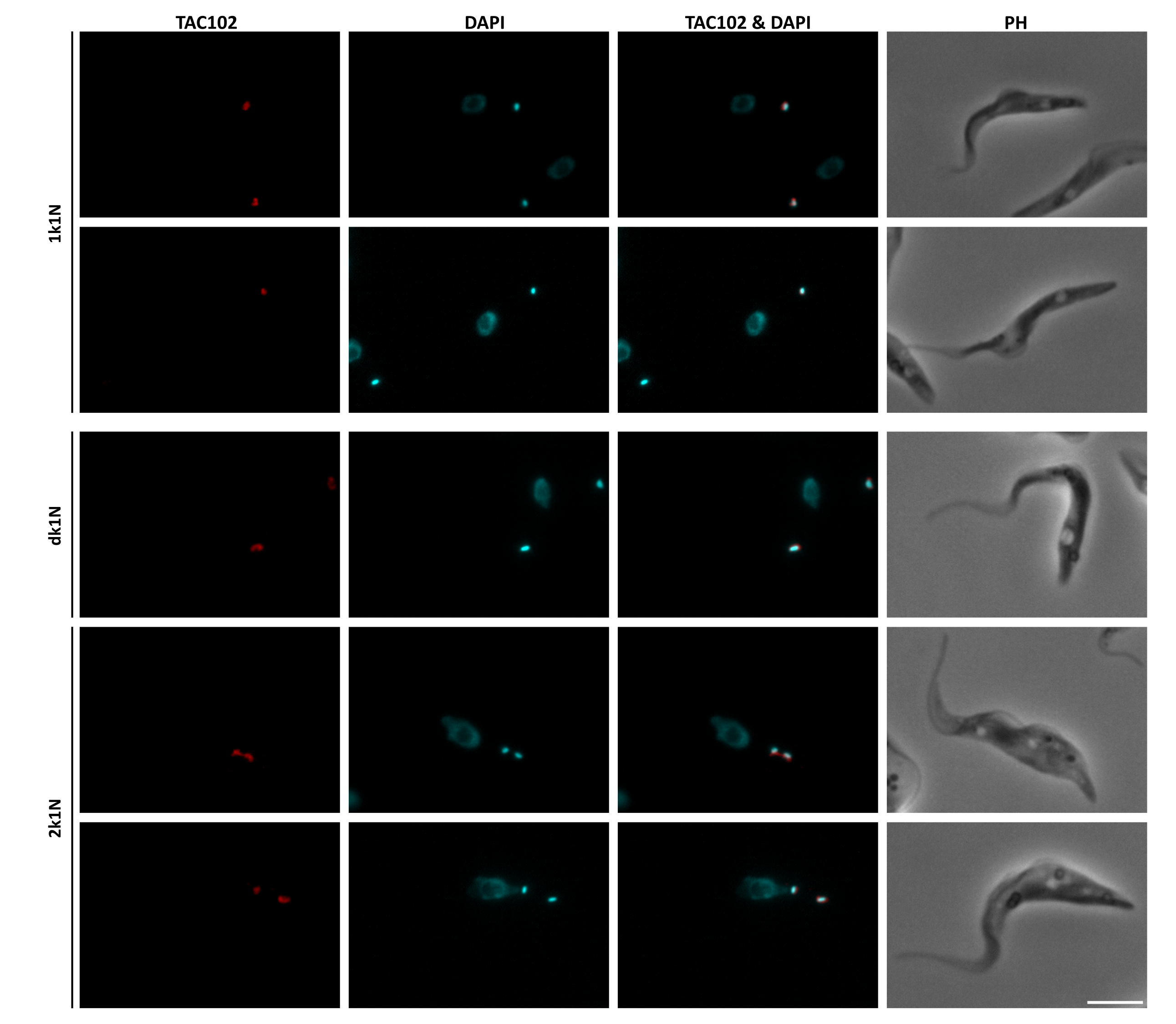

Supplement: S1 Fig — (TIF) [file ppat.1011486.s001.tif]

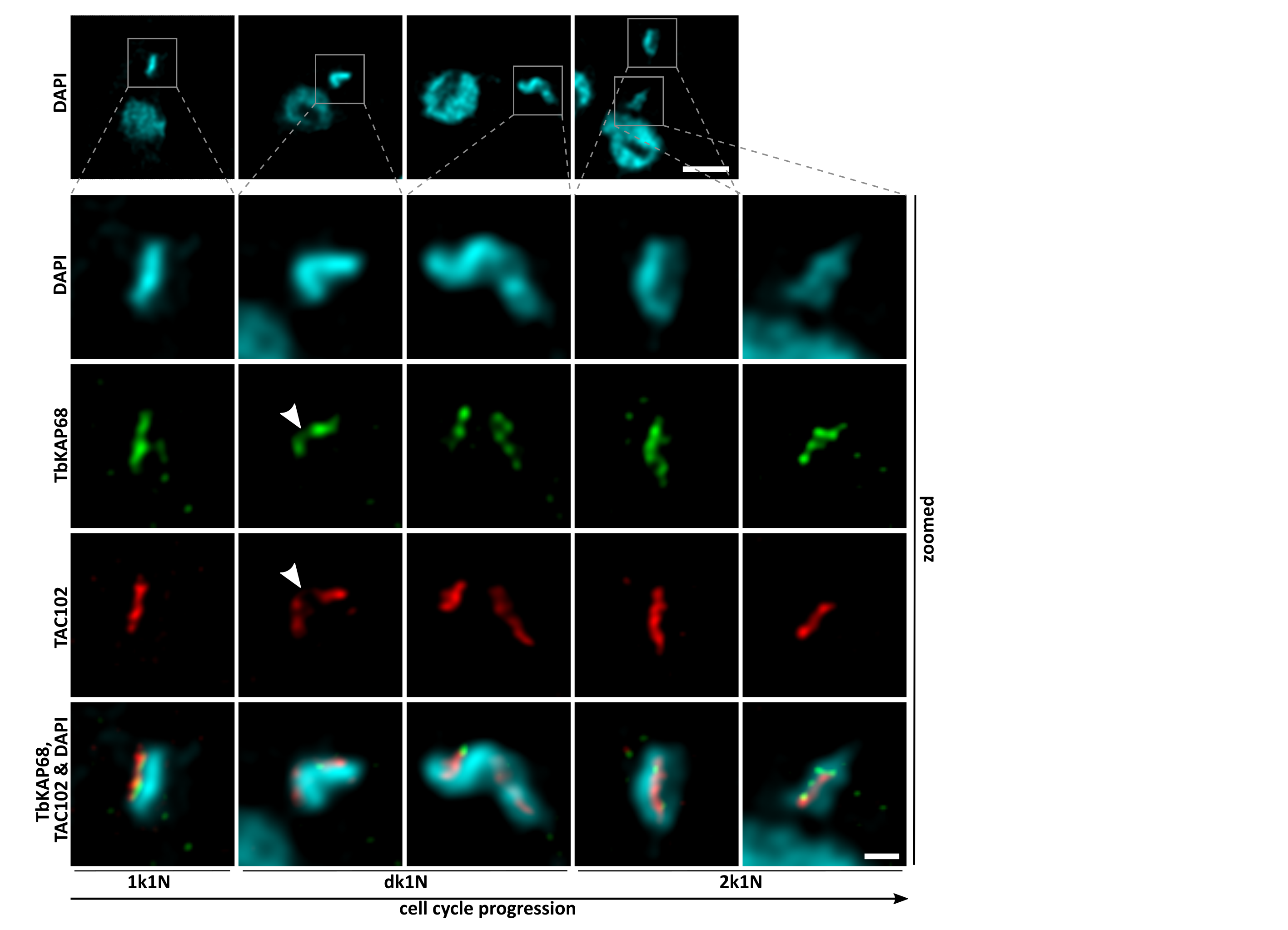

Supplement: S2 Fig — (TIF) [file ppat.1011486.s002.tif]

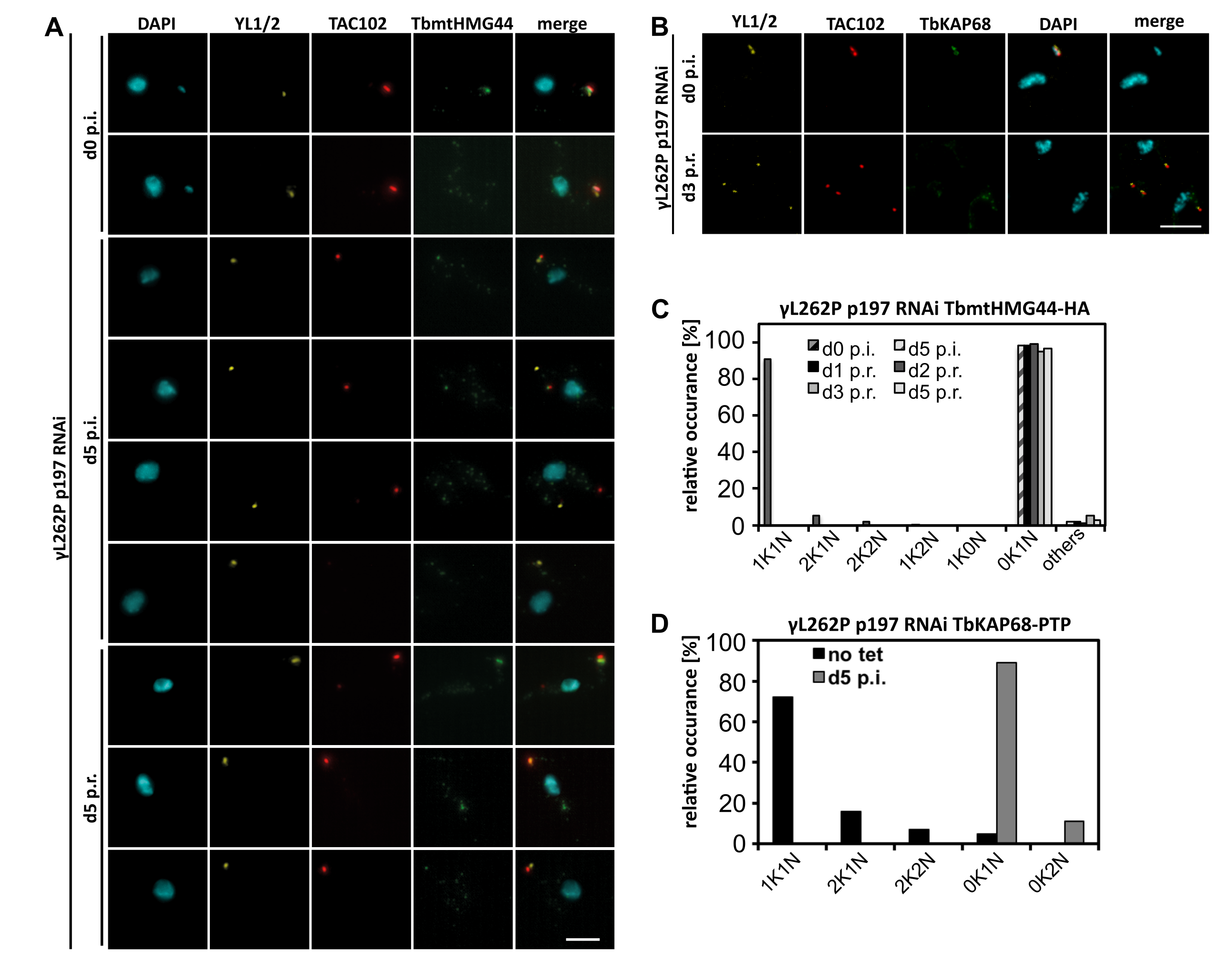

Supplement: S3 Fig — (TIF) [file ppat.1011486.s003.tif]

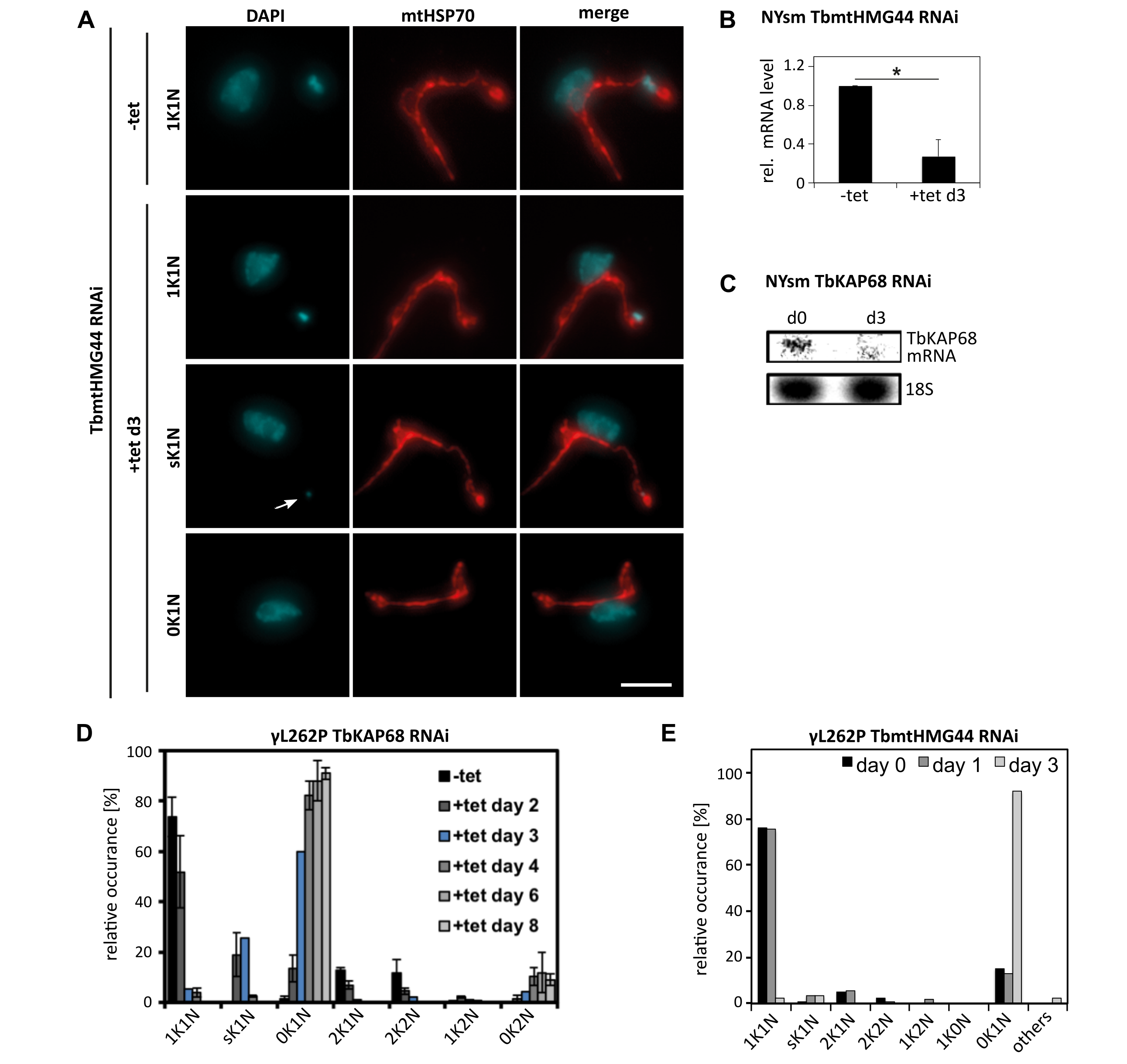

Supplement: S4 Fig — (TIF) [file ppat.1011486.s004.tif]

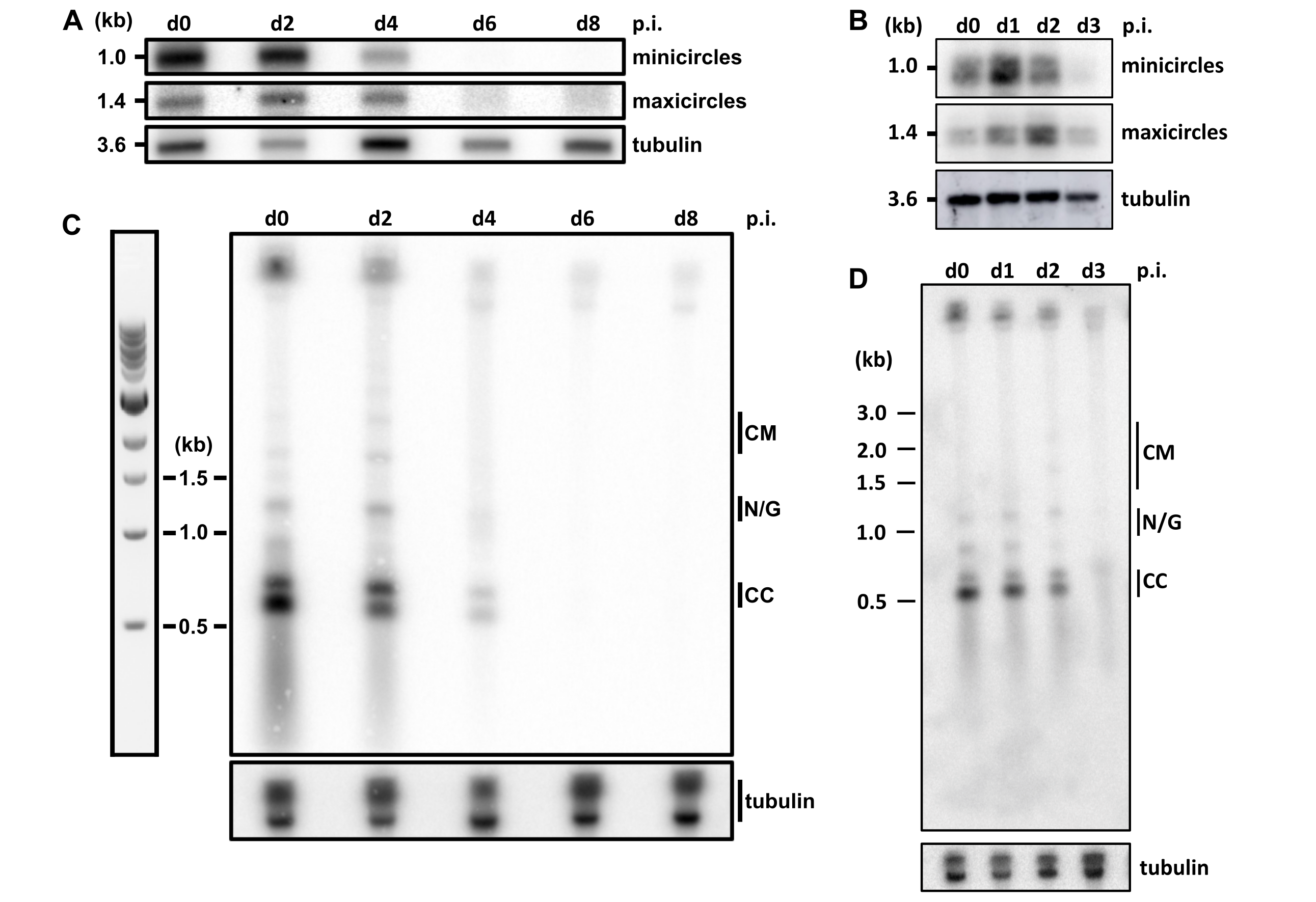

Supplement: S5 Fig — (TIF) [file ppat.1011486.s005.tif]

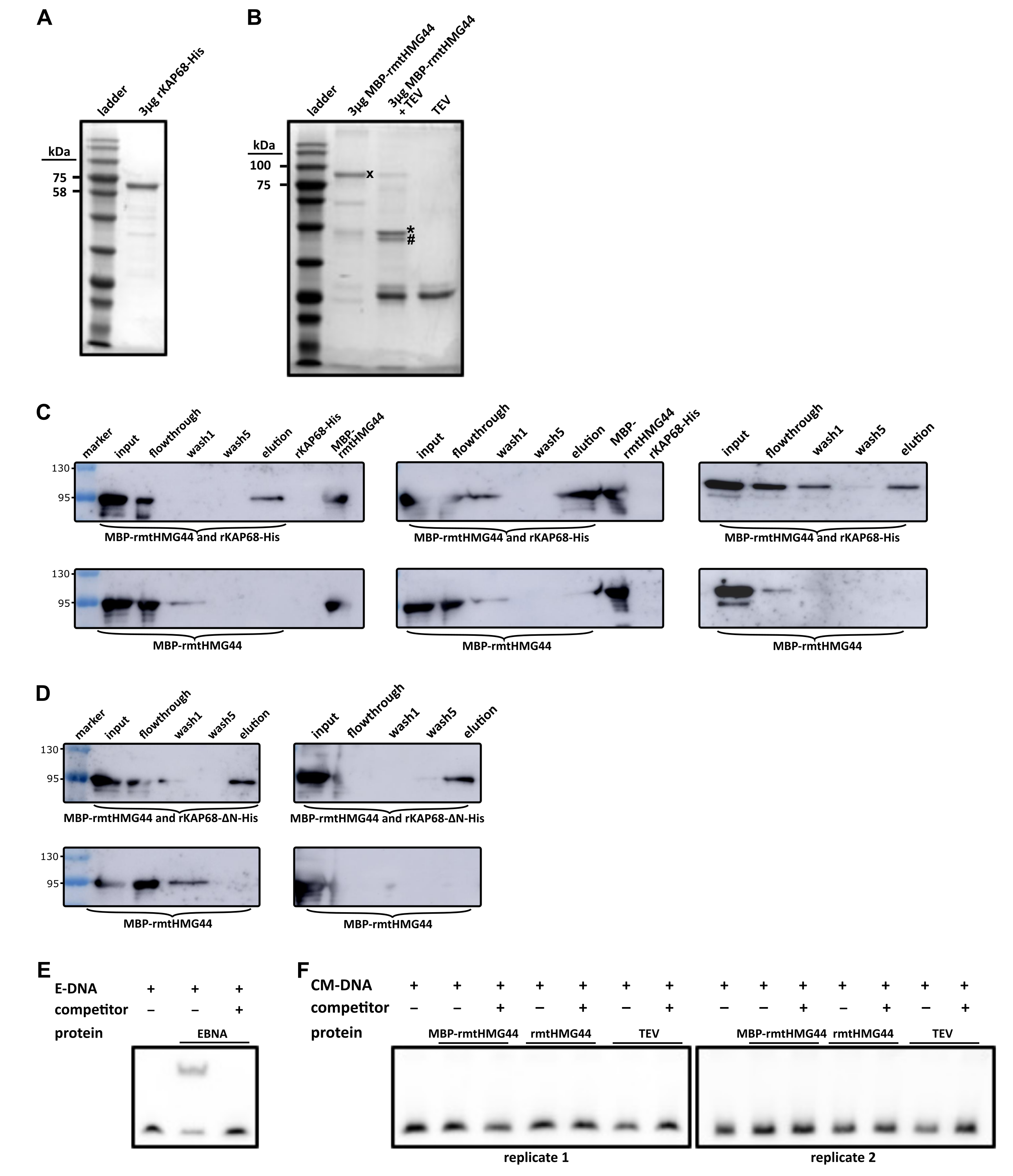

Supplement: S6 Fig — (TIF) [file ppat.1011486.s006.tif]

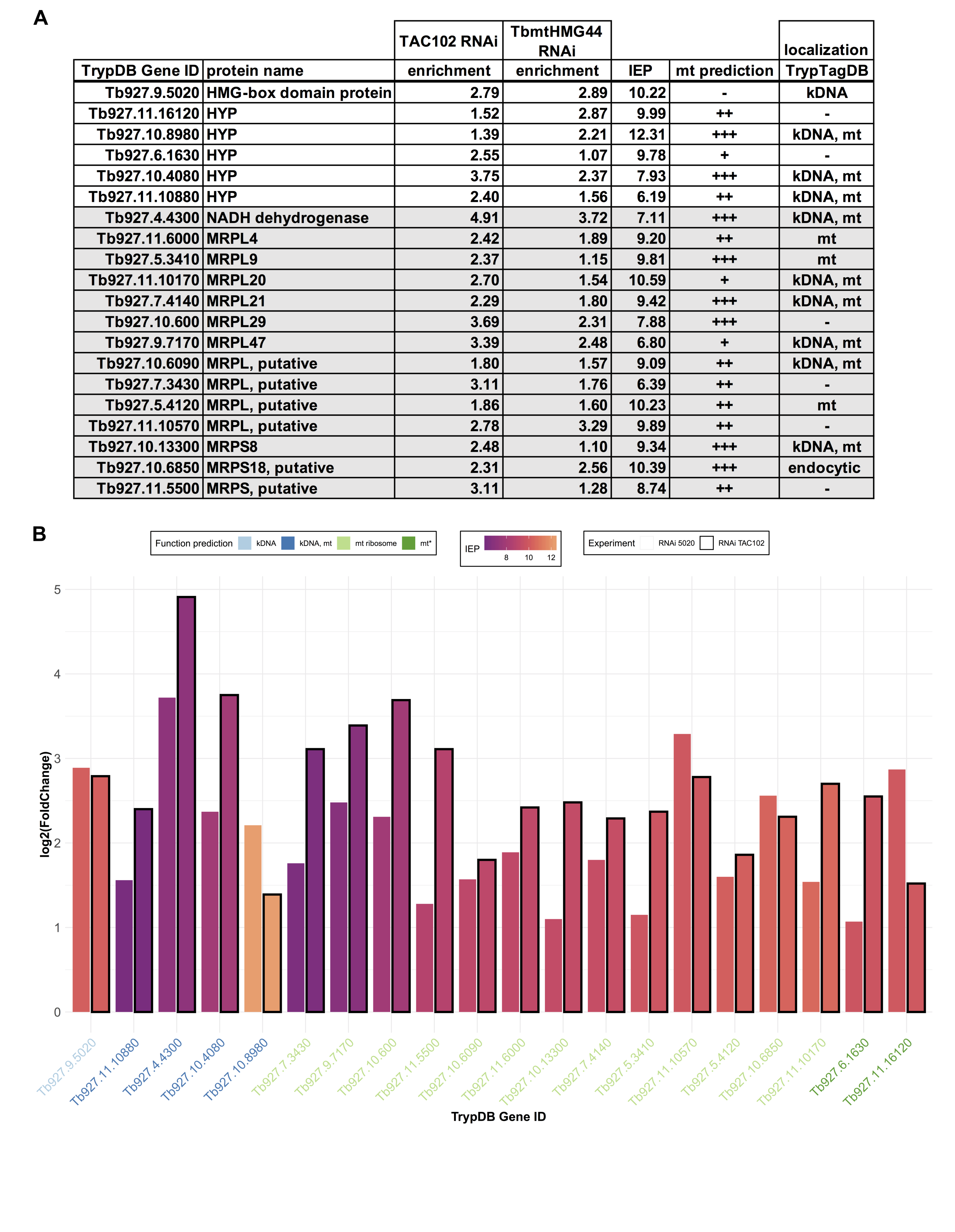

Supplement: S7 Fig — (TIF) [file ppat.1011486.s007.tif]

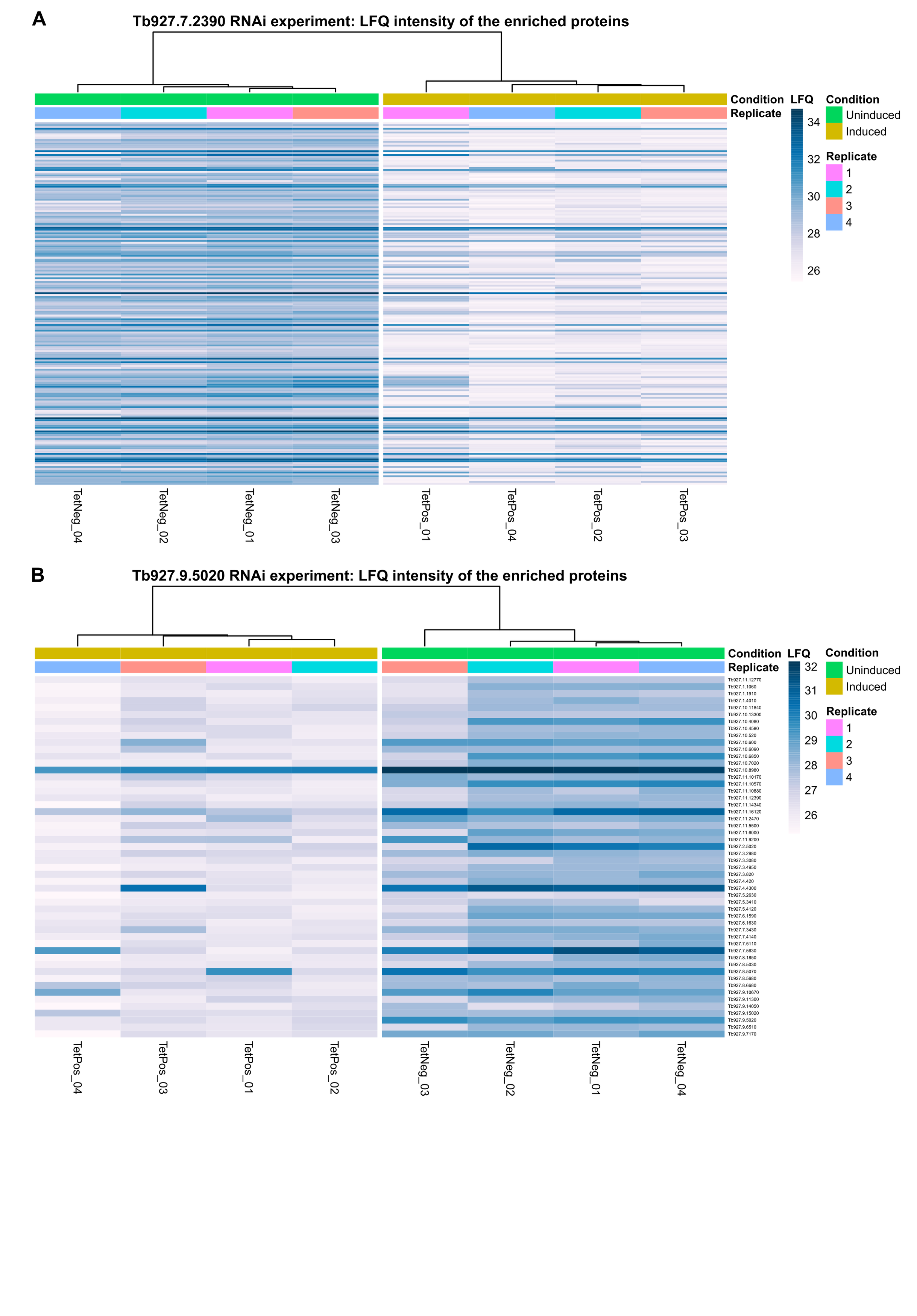

Supplement: S8 Fig — (TIF) [file ppat.1011486.s008.tif]

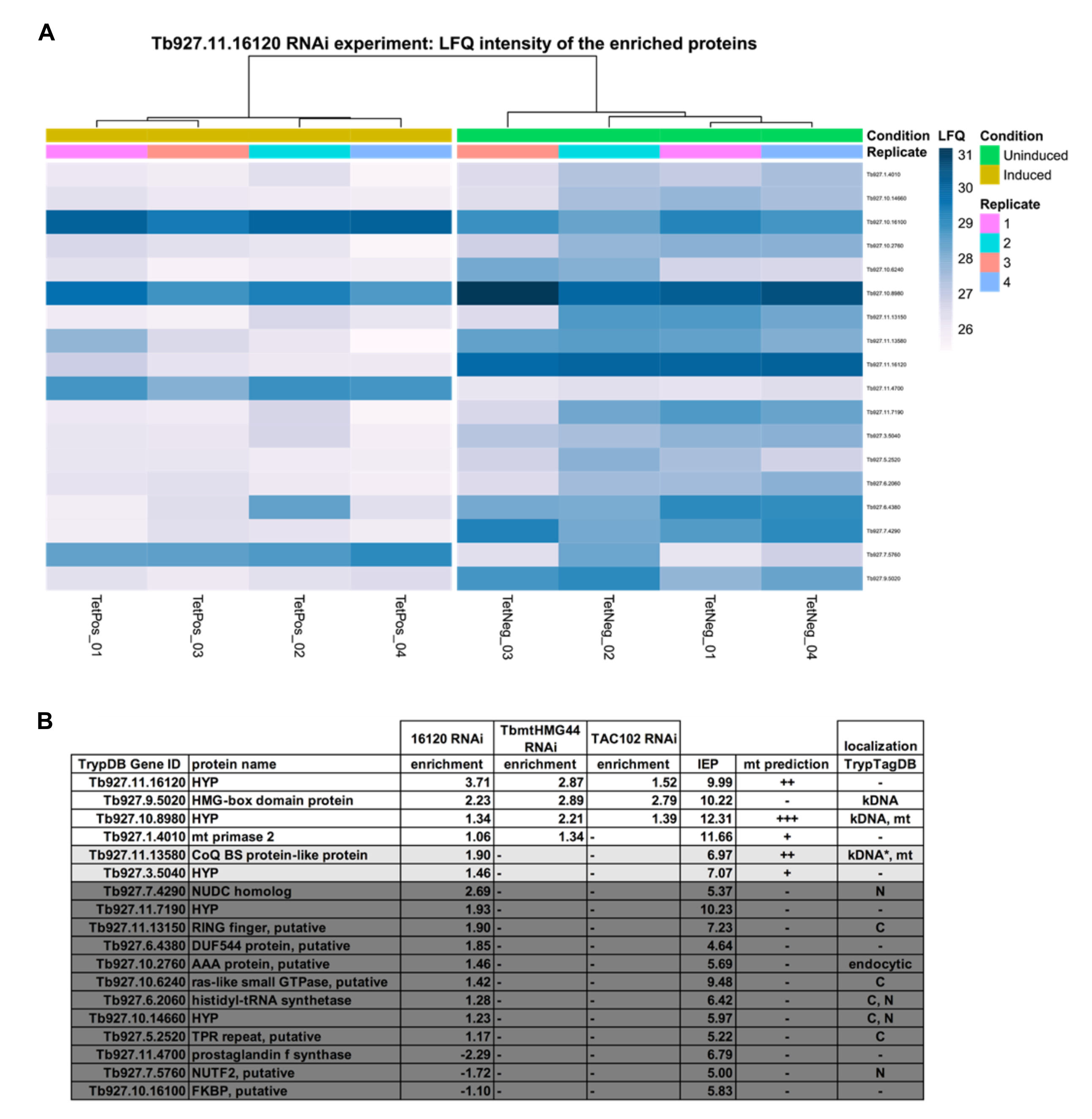

Supplement: S9 Fig — (TIF) [file ppat.1011486.s009.tif]

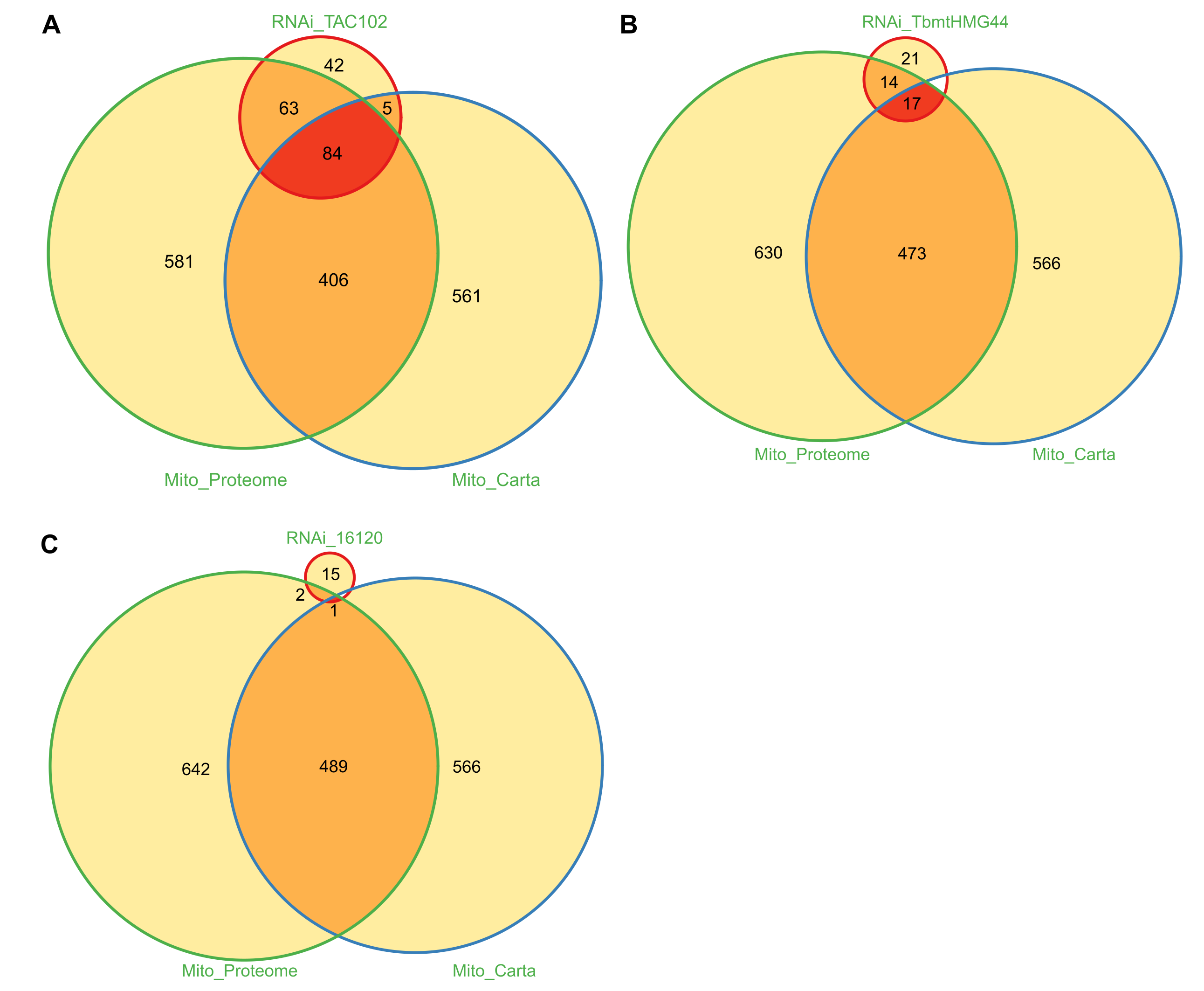

Supplement: S10 Fig — (TIF) [file ppat.1011486.s010.tif]

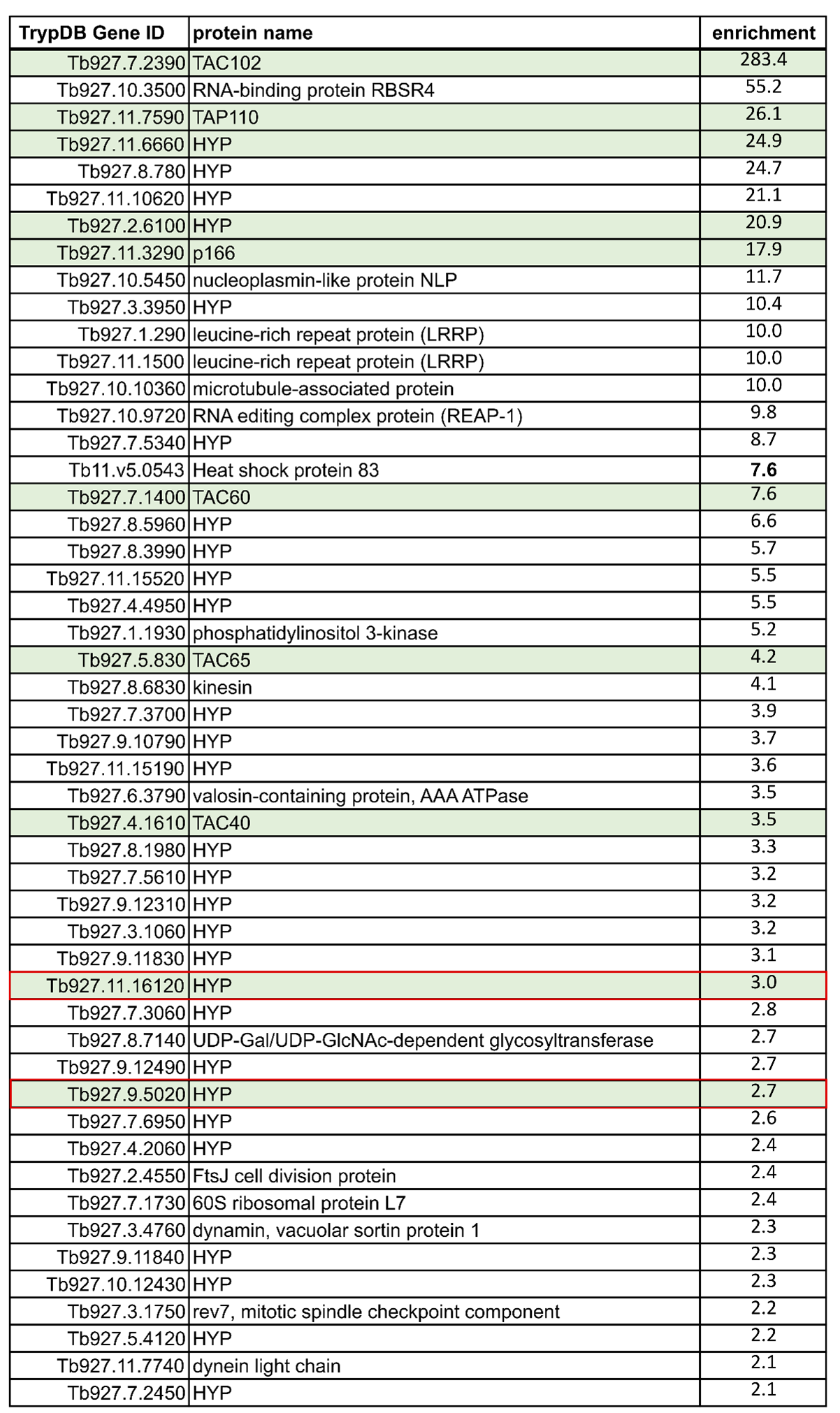

Supplement: S1 Table — (TIF) [file ppat.1011486.s011.tif]

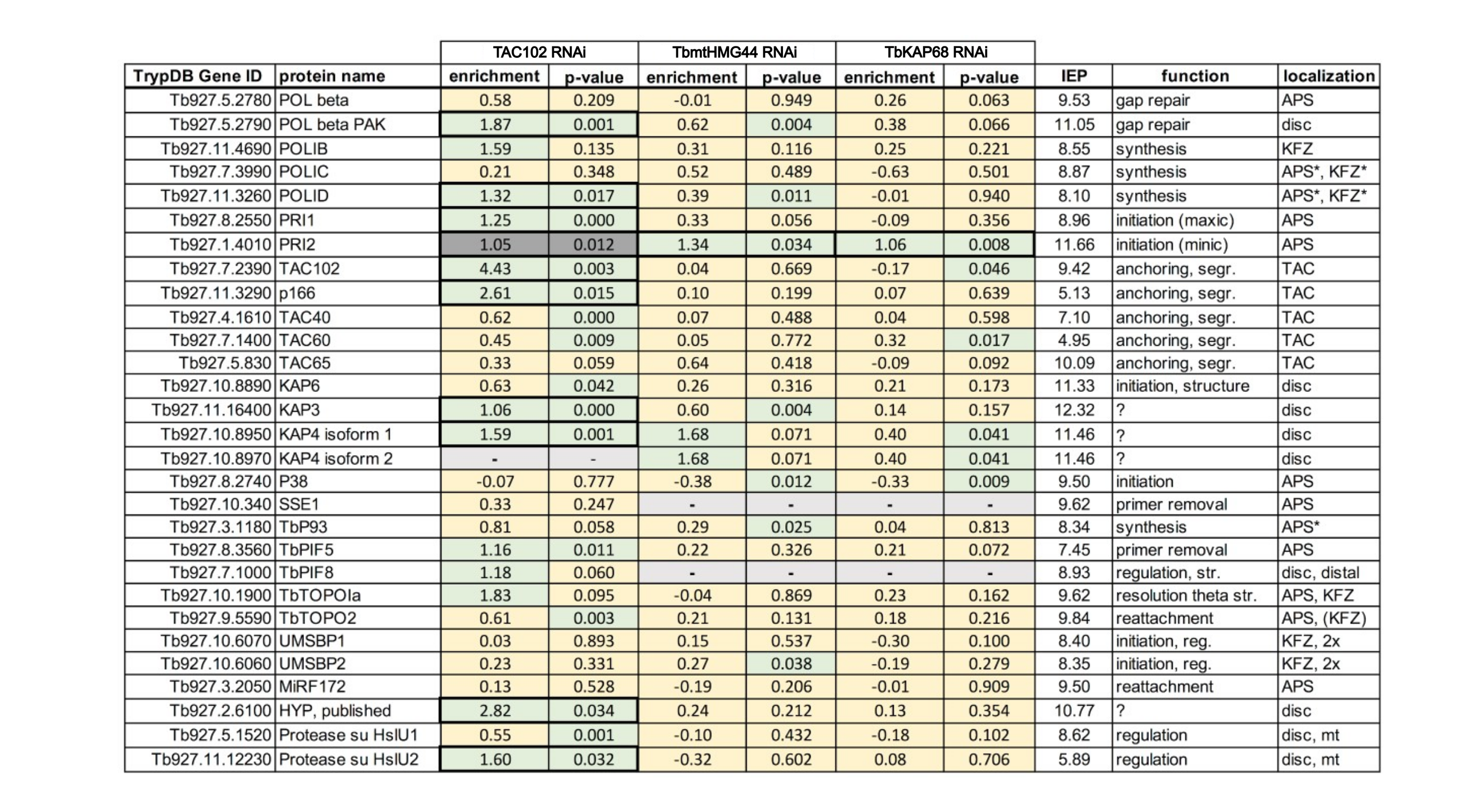

Supplement: S2 Table — (TIF) [file ppat.1011486.s012.tif]
